# Laboratory assessment of novel endophytic *Trichoderma*-based bioformulations for the biological control of sorghum leaf spot and stalk rot diseases

**DOI:** 10.1038/s41598-026-54927-w

**Published:** 2026-07-01

**Authors:** Sara Mahmoud Arafa, Khalid Mohamed Ghoneem, Allam Arafat Megahed, Yasser Mohamed Shabana

**Affiliations:** 1 Plant Pathology Department, Faculty of Agriculture, Mansura University, El-Mansoura, 35516 Egypt; 2 Seed Pathology Department, Plant Pathology Research Institute, Agricultural Research Center, Giza, 12619 Egypt; 3 Agricultural Botany Department (Plant Pathology), Faculty of Agriculture, Damietta University, New Damietta, 34517 Egypt

**Keywords:** Sorghum, Seed-borne diseases, Antifungal activity, *Trichoderma asperellum*, *Trichoderma harzianum*, Biocontrol, Biological techniques, Biotechnology, Microbiology, Plant sciences

## Abstract

Stalk rot and leaf spot, caused by *Fusarium verticillioides* and *Curvularia lunata*, respectively, are among the most destructive seed-borne fungal diseases of sorghum, resulting in substantial yield losses. In the present study, two novel endophytic strains, *Trichoderma asperellum* SEPA11A and *Trichoderma harzianum* SEPA11B, were isolated from healthy sorghum seeds and evaluated for their biocontrol potential and growth-promoting effects. Out of 39 *Trichoderma* isolates screened, the isolates 35 and 3 exhibited the highest antagonistic activity in dual culture assays, inhibiting the mycelial growth of *F. verticillioides* by 68.88% and 66.67%, and *C. lunata* by 57.77% and 59.30%, respectively. Both strains were identified based on morphological characteristics and internal transcribed spacer (ITS) rDNA sequencing and have been deposited in GenBank under accession numbers LC866760.1 and LC866759.1, and coded as SEPA11B and SEPA11A respectively. Gas chromatography–mass spectrometry (GC–MS) analysis of culture filtrates revealed 55 bioactive metabolites, while scanning electron microscopy demonstrated severe ultrastructural damage to pathogen hyphae. A granular formulation of SEPA11B and SEPA11A maintained high viability for up to 14 months under storage. These results collectively indicate that SEPA11B and SEPA11A represent promising, sustainable, and cost-effective alternatives to chemical fungicides for integrated management of stalk rot and leaf spot diseases, with the added as a potential benefit of enhancing sorghum growth and productivity. This unique trait makes seed-derived endophytic *Trichoderma* isolates promising for effective, persistent biofungicide development. Adapted to the seed-soil interface, they can colonize the rhizosphere, outcompete pathogens, and remain active under field conditions. Thus, they are strong candidates for sustainable biofungicides, pending greenhouse/field validation and large-scale production optimization.

**Supplementary Information:**

The online version contains supplementary material available at 10.1038/s41598-026-54927-w.

## Introduction

Sorghum (*Sorghum bicolor* L.) is a globally important cereal crop, ranking fifth among cereal grains worldwide^1^. Beyond its critical role in ensuring food security, sorghum is also a valuable resource for animal feed and biodiesel production. The crop is primarily cultivated under dry and semi-arid conditions and is widely grown across Africa, Asia, and the Americas. Africa alone contributes approximately 20 million tons to the global annual harvest. However, the increasing prevalence and severity of sorghum diseases are not occurring in a vacuum; they are being intensified by the growing pressures of climate change. Environmental stressors—such as prolonged drought and extreme temperature fluctuations—can weaken the plant’s natural defense mechanisms, making it more vulnerable to infection. This climate-induced vulnerability creates opportunities for pathogens to thrive, transforming what were once minor diseases into major economic threats^2^. According to FAOSTAT^3^, Egypt cultivates 150,000 hectares of sorghum, producing approximately 780,000 tons yearly. Under Egypt Vision 2030, expansion of drought-tolerant crops such as sorghum is a national priority to address climate change challenges, achieve food security, and promote sustainable agriculture. Data from the Foreign Agricultural Service (2025) indicate that Asyut, Sohag, and Fayoum governorates collectively account for more than 80% of Egypt’s sorghum production.

Sorghum is susceptible to more than 50 diseases, the majority of which are seed-borne, with fungal pathogens representing the most significant constraint to production^4^. Seed-borne fungal diseases significantly reduce sorghum yield and quality, impair germplasm and seed production, and limit domestic and international trade^5^. Seed treatment is considered the most effective approach for controlling these pathogens^6^. Seed treatment is considered among the most effective approaches for managing seed-borne pathogens, as it protects germinating seedlings during their most vulnerable stage. This localized protection enhances germination, promotes early plant vigor, and reduces the need for extensive fungicide applications later in crop development^7–9^. The reported yield loss of 12–55% for *Curvularia lunata* (*Curvularia* leaf spot) in sorghum reflects region-specific conditions, particularly in tropical and subtropical areas with high humidity and rainfall, rather than a uniform global average^10–12^. In Egypt, *C. lunata* has been identified as one of the major fungi affecting sorghum grain genotypes under both storage and field conditions^13,14^. Recently, it has emerged as a significant pathogen responsible for sorghum leaf spot and grain mold, adversely impacting both grain quality and yield^15^. *Fusarium* stalk rot, caused by several *Fusarium* spp., is another major disease of sorghum. The pathogen colonizes roots and stalk tissues, causing vascular discoloration, cortical tissue degradation, and impaired water and nutrient translocation, often resulting in plant lodging under severe infection^16^. *Fusarium* mycotoxins, particularly fumonisins, zearalenone, and deoxynivalenol (DON), frequently contaminate sorghum grains, posing significant health risks to both humans and livestock. Humans are exposed primarily through consumption of contaminated sorghum-based food products, while livestock may be affected via contaminated feed, leading to chronic health issues such as esophageal cancer, neural tube defects, immune suppression, and reduced productivity^17,18^.

Chemical fungicides remain a primary management strategy but raise concerns regarding environmental contamination, non-target effects on beneficial microbiota, and risks to human and animal health^19^. Additionally, the emergence of fungicide-resistant pathogen populations has intensified the search for eco-friendly alternatives^20^. Biological control represents a sustainable, non-toxic, and cost-effective alternative, often reducing management costs by up to 50% compared with chemical fungicides^21,22^. Moreover, biocontrol agents (BCAs) enhance plant growth, induce systemic resistance, and improve yield^23^. Numerous studies have demonstrated the efficacy of biocontrol agents in seed treatment against a wide range of seed-borne fungi^24^.

Among biocontrol agents, *Trichoderma* species are the most effective antagonists against several plant pathogens, including fungi. *Trichoderma* functions as a biocontrol agent through two primary mechanisms: Direct antagonism and induced systemic resistance (ISR). Direct antagonism occurs when the fungus physically or chemically attacks pathogens via mycoparasitism (coiling and enzyme degradation), antibiosis (toxin production), and competition for nutrients. In contrast, ISR involves colonization of plant tissues that primes the plant’s own defenses, enhancing production of phenolics, strengthening cell walls, and activating defense pathways without direct contact with the pathogen^25,26^. *Trichoderma* spp. rapidly colonizes the rhizosphere, forming a protective barrier against pathogen invasion^27^, and have been shown to be effective against various pathogens, including *Sclerotinia sclerotiorum*, *S. rolfsii*, *Macrophomina phaseolina*, *Rhizoctonia solani*,* Pythium* spp., *Phytophthora* spp., *Fusarium* spp. and *Curvularia *spp.^28–35^. Additionally, *Trichoderma* produces secondary metabolites that contribute to pathogen suppression, plant growth promotion, systemic resistance induction, and control pest^36^. *Trichoderma* biocontrol agents are commonly produced using cost-effective solid-state (SSF) and liquid (LSF) fermentation, with carrier materials such as wheat bran, semolina, or vermiculite used to stabilize the formulations. Solid-state methods often provide higher viability, whereas liquid methods allow efficient large-scale production. Additionally, new technologies, including encapsulation and the use of carriers, enhance shelf life and improve field efficacy^37,38^. Endophytic *Trichoderma* isolated from seeds represents a novel source compared to rhizospheric isolates due to its unique ecological niche and lifestyle. Unlike soil-dwelling rhizospheric strains, which are generally opportunistic, seed endophytes are specialized to colonize internal plant tissues, allowing them to persist within the host, transmit vertically through seeds, and provide systemic protection against pathogens from the earliest stages of plant development. This internal colonization often confers enhanced tolerance to environmental stresses such as drought, heat, and microbial competition, and can result in greater antagonistic activity against seed-borne pathogens^39,40^. The exploitation of seed-derived endophytic *Trichoderma* is therefore particularly promising for the development of biofungicides, as these strains are already adapted to the internal seed environment, ensuring effective colonization, long-term persistence, and consistent biocontrol activity when applied to crops.

Commercial formulations of *T. harzianum* and *T. viride* are currently used against more than 70 soil-borne and 18 foliar pathogens affecting over 87 crops^39^. The success of *Trichoderma*-based products is attributed to their rhizosphere competence, broad-spectrum activity, and compatibility with other BCAs^41^. The shelf life of formulations varies with storage conditions and carrier material, ranging from 3 to 4 months in kaolin-based carriers to over 18 months in coffee-husk-based formulations^42^. The literature highlights a need for more comprehensive and current studies on seed-borne pathogens specifically affecting sorghum in Egypt. Furthermore, there is limited information on locally adapted *Trichoderma* strains for effective biocontrol of these pathogens. This study addresses these gaps by isolating, characterizing, and evaluating endophytic *Trichoderma* strains from Egyptian sorghum seeds, providing both scientific insights and practical strategies for managing seed-borne fungal diseases^43^.

This study addresses key research gaps by focusing on seed-derived endophytic *Trichoderma* strains from sorghum in Egypt, which offer unique advantages over conventional rhizospheric strains due to their internal colonization, vertical transmission, and enhanced antagonism against seed-borne pathogens. While previous research has largely relied on in vitro antagonism without linking metabolite profiles to biocontrol function, and has seldom developed granular formulations from locally adapted strains, this study combines molecular characterization, metabolite profiling, and formulation development to create a stable, targeted granular biofungicide for sustainable management of sorghum leaf spot and stalk rot.

## Results

### Distribution of sorghum endophytic seed-borne mycobiota

**Fig. 1:**
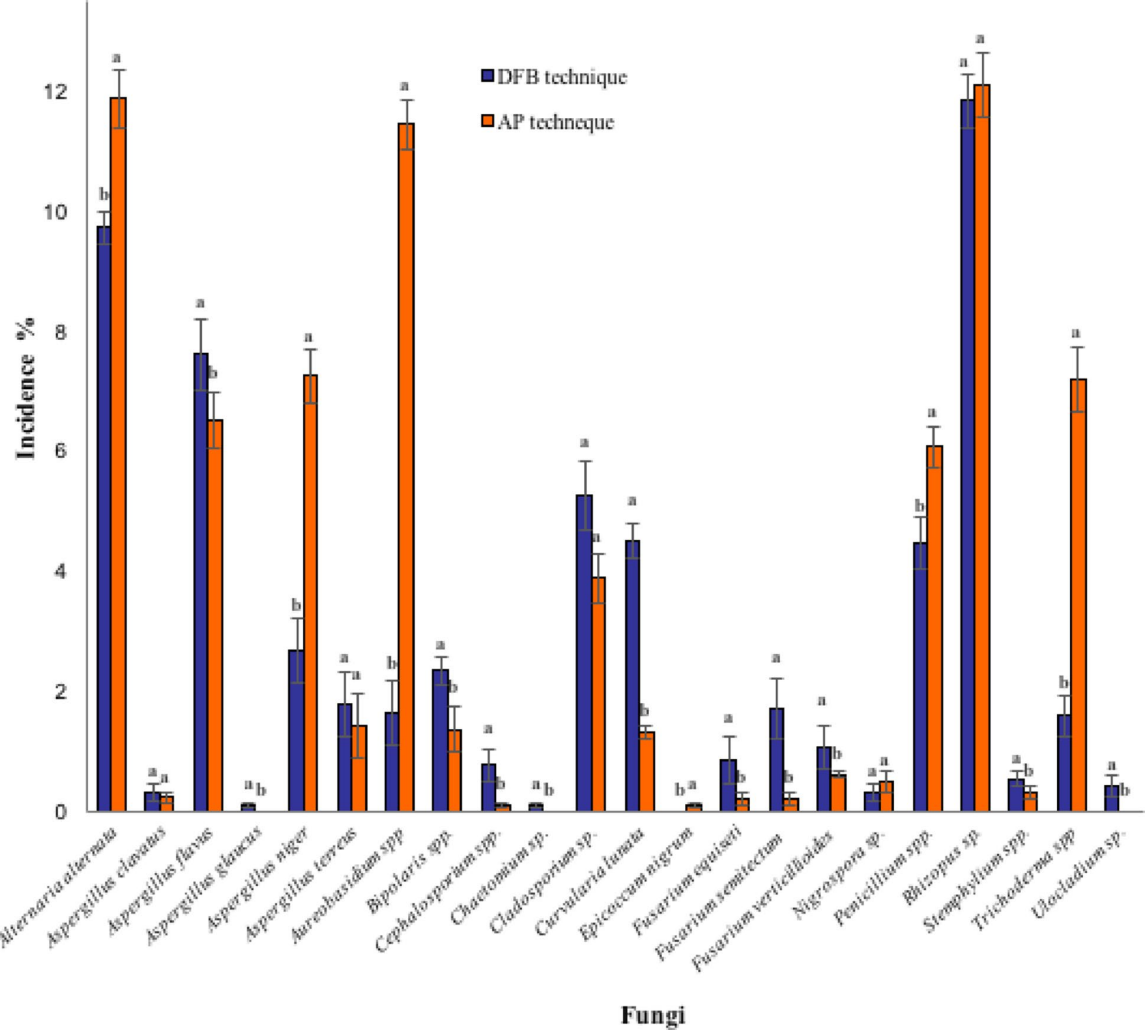
Illustrating the frequency distribution (mean ± SE) of 22 endophytic seed-borne fungal taxa isolated from sorghum seeds collected from six sorghum-growing governorates in Egypt during the 2023 cropping season. Frequencies were determined using both the deep-freezing blotter (DFB) and agar plate (AP) techniques.

**Fig. 2:**
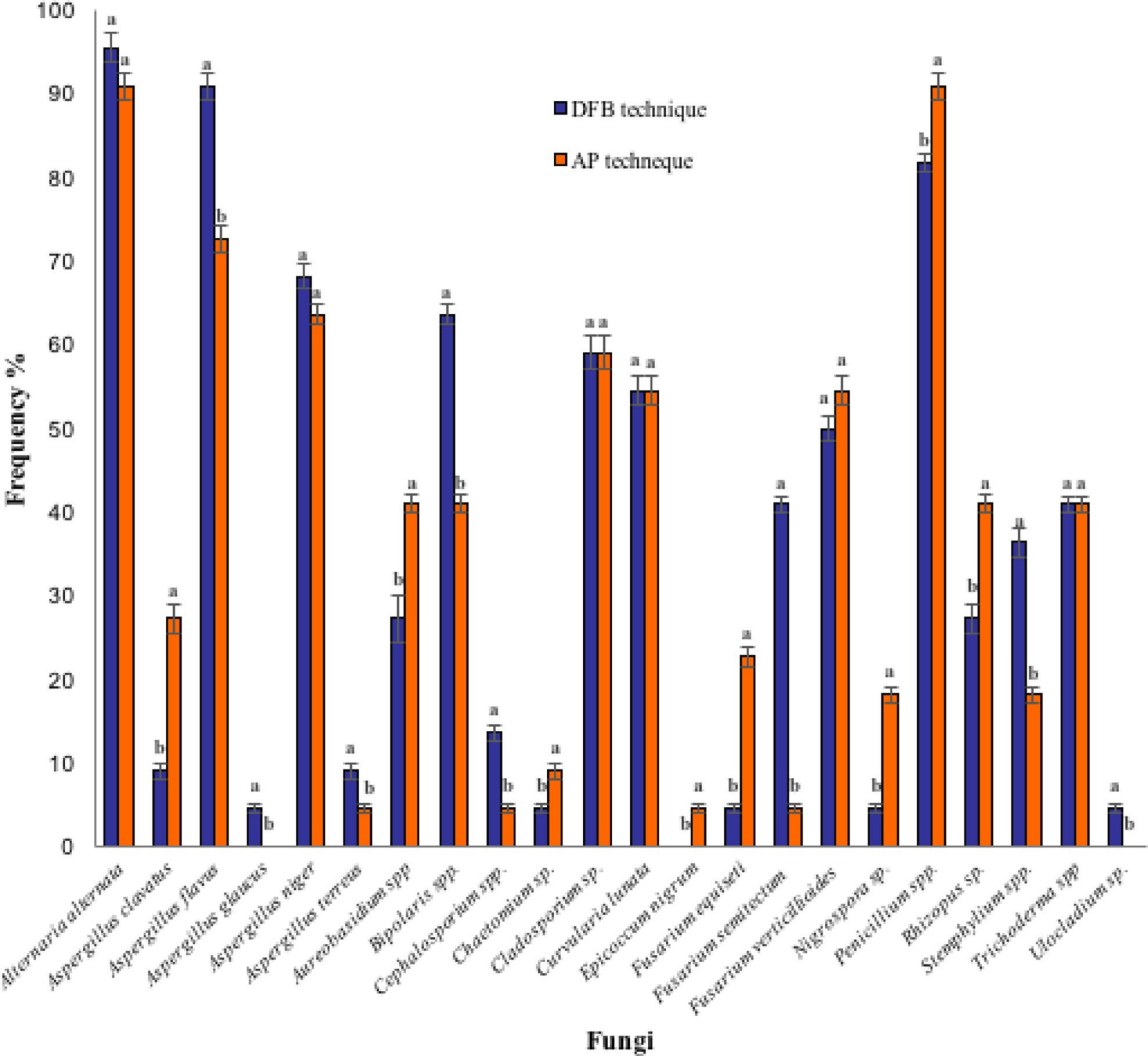
Depicting the mean incidence (% of seeds infected) (mean ± SE) of 22 endophytic seed-borne fungal taxa recovered from sorghum seeds collected from six sorghum-growing governorates in Egypt during the 2023 cropping season. Incidences were determined using both the deep-freezing blotter (DFB) and agar plate (AP) techniques.

Seed-borne fungal endophytes were detected and identified from healthy-looking sorghum seed samples collected across six major sorghum-growing governorates in Egypt during the 2023 cropping season. A total of 22 composite seed samples were analyzed using both the agar plate (AP) and deep-freezing blotter (DFB) techniques. These analyses revealed 16 fungal genera encompassing 22 endophytic species (Figs. 1 and 2). Among the recovered taxa, *Alternaria alternata*, *Penicillium* spp., and *Aspergillus flavus* were the most frequently detected endophytic fungi across all sorghum-growing regions, followed by *Aspergillus niger*, *Bipolaris* spp., *Cladosporium* spp., *Curvularia lunata*, *Fusarium verticillioides*, and *Trichoderma* spp. Less frequent species included *Aspergillus terreus*, *Epicoccum nigrum*, and *Fusarium incarnatum*. Detailed frequency and incidence data are summarized in Figs. (1 and 2). In contrast, *Aspergillus terreus*, *Cephalosporium* spp., *Epicoccum nigrum*, and *Fusarium incarnatum* (synonym: *F. semitectum*) were the least frequently detected (4.55 each with the AP), while *Aspergillus glaucus*, *Chaetomium* sp., *Fusarium equiseti*, *Nigrospora* spp., and *Ulocladium* spp. recorded the lowest frequency with DFB. Notably, *E. nigrum* was exclusively detected with AP, whereas *A. glaucus* and *Ulocladium* sp. were exclusively detected with DFB (Fig. 1). Several isolates of the pathogenic fungus *C. lunata* were consistently detected, with a frequency of 54.55% using both AP and DFB techniques. In contrast, isolates of *F. verticillioides* exhibited frequencies of 54.55% with AP and 50.00% with DFB. *Cephalosporium* spp. exhibited a comparatively lower frequency rate, 4.55% (AP) and 13.63% (DFB). Among potential biocontrol fungi, *Trichoderma* spp. was the only taxon consistently detected by both AP and DFB methods, with a frequency of 40.91% (Fig. 2). Differences in fungal detection between the agar plate (AP) and deep-freezing blotter (DFB) methods were observed. The AP technique generally detected a broader range of fungi, whereas the DFB method facilitated the identification of certain slower-growing pathogens. A detailed explanation of the methodological sensitivity differences is provided in the discussion section.

### Dual culture antagonism assay

The antagonistic potential of 39 isolates of *Trichoderma* spp. against *F. verticillioides* (SOR2) and *C. lunata* (SOR7) was evaluated under in vitro conditions. Results revealed that most displayed marked inhibitory effects on mycelial growth, with growth reduction percentages ranging between 35.53% and 73.33% (Table 1).

**Table 1:** Effect of *Trichoderma* spp. isolates (TI) on radial growth inhibition (%) of *Fusarium verticillioides* (SOR2) and *Curvularia lunata* (SOR7) in dual culture assays.

TI	Growth reduction (%)		AG	TI	Growth reduction (%)		AG
	FV-SOR2	CL-SOR7			FV-SOR2	CL-SOR7	
**1**	53.32e-k	51.20 g-i	1	**21**	55.60 d-g	50.40 hi	1
**2**	55.55d-g	52.60 e-h	1	**22**	55.73 d-f	53.47 d-h	1
**3**	66.67ab	59.30 b	1	**23**	55.93 d-f	48.12 ij	1
**4**	52.53f-l	52.59 e-h	1	**24**	57.27 de	53.31 e-h	1
**5**	46.67mn	51.23 g-i	1	**25**	51.20 g-l	53.36 e-h	1
**6**	54.87e-j	55.57 c-e	2	**26**	48.67 l-n	57.80 bc	1
**7**	51.20 g-l	52.57 e-h	1	**27**	55.43 e-g	54.96 c-f	1
**8**	55.60d-g	50.47 hi	1	**28**	53.30 e-k	55.63 c-e	1
**9**	50.37 j-m	52.59 e-h	1	**29**	44.57 n	44.50 k	1
**10**	66.66 ab	48.20ij	1	**30**	60.00 cd	59.70 b	1
**11**	55.60 d-g	52.61 e-h	1	**31**	48.90 k-n	53.31 e-h	1
**12**	57.75 de	51.13 g-i	1	**32**	55.43 e-g	52.61 e-h	1
**13**	54.80 e-j	55.67 c-e	1	**33**	62.50 bc	51.17 g-i	1
**14**	48.88 k-n	35.53 l	1	**34**	54.81 e-j	54.17 d-g	1
**15**	55.57 d-g	51.88 f-h	1	**35**	68.88 a	57.77 bc	1
**16**	53.33 e-k	73.33 a	1	**36**	50.50 h-m	46.66 jk	1
**17**	54.90 e-i	54.96 c-f	1	**37**	54.07 e-j	57.07 b-d	1
**18**	66.70 ab	46.67 jk	1	**38**	54.97 e-h	50.37 hi	1
**19**	54.90 e-i	51.27 g-i	1	**39**	60.03 cd	55.55 c-e	1
**20**	50.43 i-m	55.67 c-e	1				
*P* value	< 0.0001						

FV-SOR2 = *Fusarium verticillioides* sorghum isolate 2, CL-SOR7 = *Curvularia lunata* sorghum isolate 7, AG = Antagonism grade. Mean values followed by different letters within the same column are significantly different according to Tukey’s test at *P* ≤ 0.05 (*n* = 3).

**Fig. 3:**
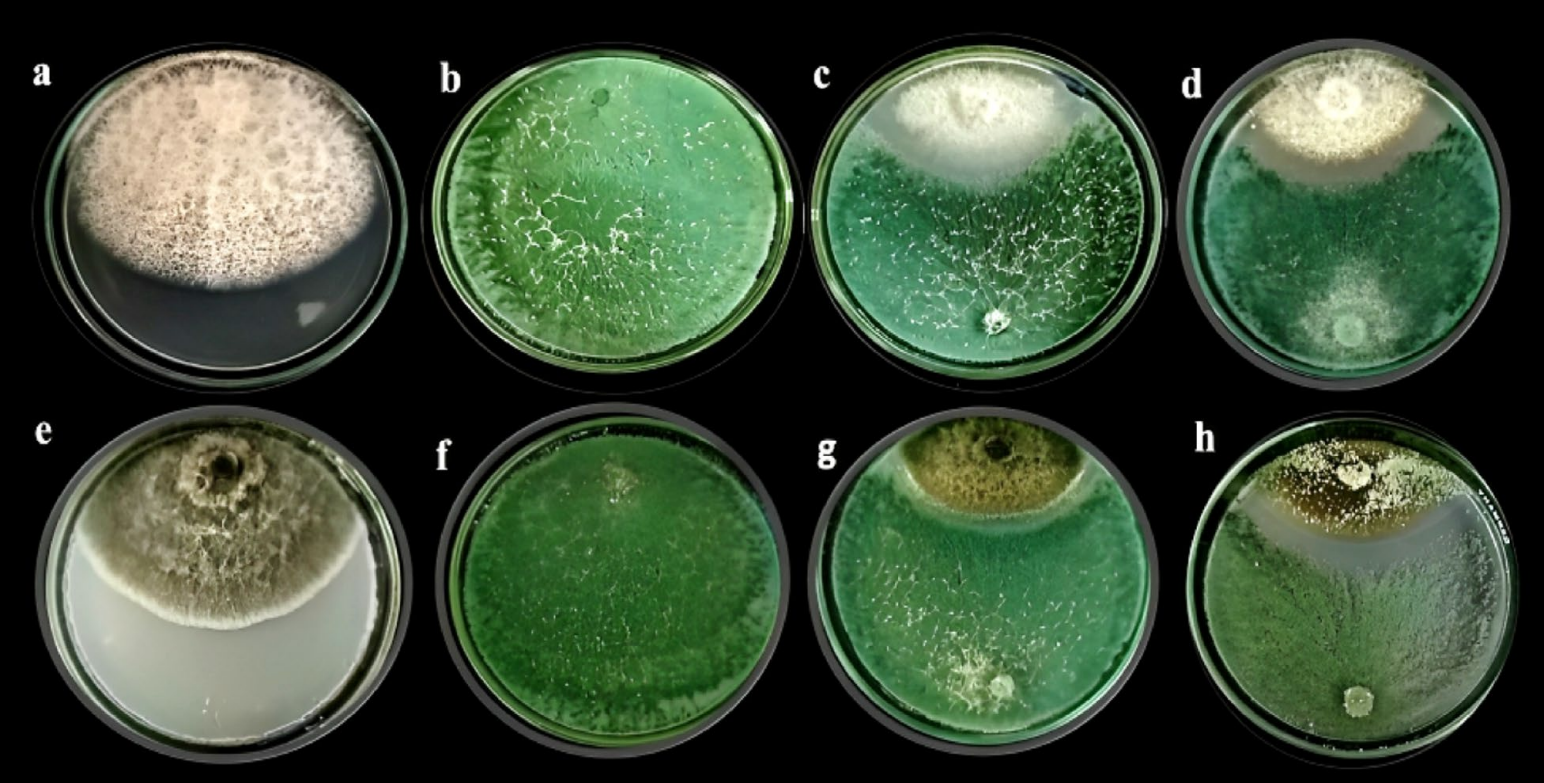
In vitro anti-fungal activities of *Trichoderma* isolate 3 and 35 against *Fusarium verticillioides* (SOR2) and *Curvularia lunata* (SOR7). **A** = Only *F. verticillioides* (SOR2) control; **B** = Only *Trichoderma* isolate 3 control; **C** = *Trichoderma* isolate 3 + *F. verticillioides*; **D** = *Trichoderma* isolate 35 + *F. verticillioides*; **E** = Only *C. lunata* (SOR7) control; **F** = *Trichoderma* isolate 35 control; **G** = *Trichoderma* isolate 3 + *C. lunata* (SOR7); **H** = *Trichoderma* isolate 35 + *C. lunata* (SOR7).

Among these, isolates 3 and 35 of *Trichoderma* spp. exhibited consistently high antifungal activity, reducing the radial growth of *F. verticillioides* by 66.67% and 68.88%, and *C. lunata* by 59.30% and 57.77%, respectively. *Trichoderma* isolate 10 followed closely, achieving 66.66% inhibition against *F. verticillioides* (Table 1; Fig. 3).

Upon extended incubation for an additional eight days, both *Trichoderma* isolate 3 and *Trichoderma* isolate 35 completely overgrew and suppressed pathogen colonies. Light microscopy observations revealed pronounced mycoparasitism, characterized by fragmented, vacuolated, and disrupted pathogen hyphae compared to the untreated controls. Based on their superior antagonistic performance, isolates *Trichoderma* isolate 3 and *Trichoderma* isolate 35 were selected for subsequent morphological, molecular, and biochemical characterization.

### Cultural and morphological characteristics of *Trichoderma* spp.

**Fig. 4:**
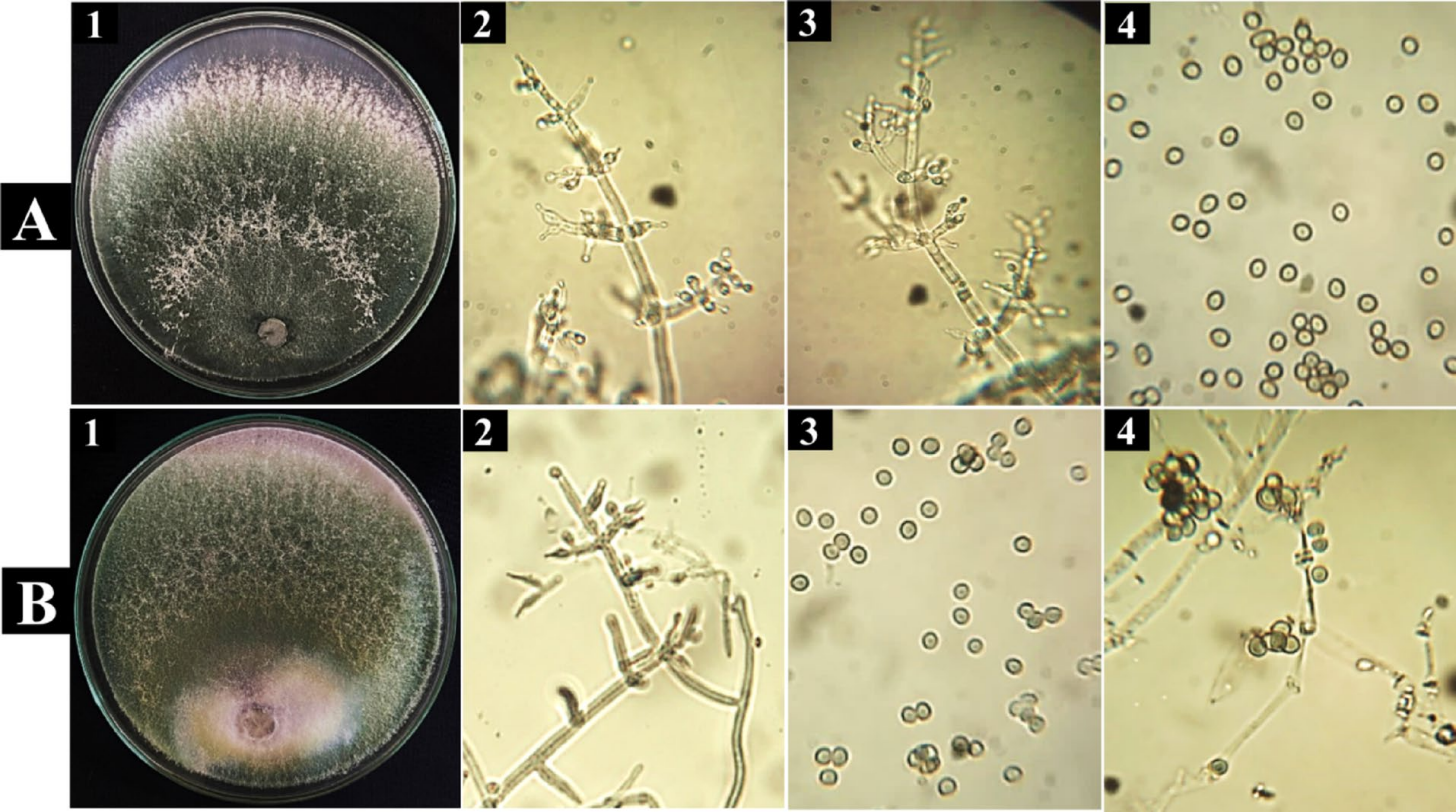
**A**, Cultural and morphological characteristics of *Trichoderma* isolate 3 as *T. asperellum*; **A,** colony observed on a PDA plate after 5 days of incubation at 25 ± 2 °C; 1–3, Micrographs showing conidiophores and phialides; 4, conidia. **B,** Growth and morphological characteristics of *Trichoderma* isolate 35 as *T. harzianum* SEP11B. 1, Colony observed on a PDA plate after 5 days of incubation at 25 ± 2 °C; 2, Micrographs illustrating conidiophores and phialides; 3, conidia; 4, chlamydospores. Bars = 10 μm.

On PDA, *Trichoderma* isolates 3 was culturally and morphologically identified as *T. asperellum* depending on that it produced colonies reaching 50 mm in diameter with dark green conidial regions. Microscopic examination revealed a well-developed main axis with several lateral branches. Conidiogenous cells (phialides) were arranged in whorls of two to four, measuring 7–14 μm long and 3–4.5 μm wide. Conidia were subglobose to ovoid measuring 3.5–4.6 μm, and chlamydospores were not observed (Fig. 4A).

The *Trichoderma* isolates 35 was identified as *T. harzianum* similarly for it formed colonies up to 55 mm in diameter with a gray-green conidial region. Microscopically, short main conidiophores were observed, each bearing 3–5 divergent ampulliform phialides measuring 6–7.5 μm in length and 3.2–3.5 μm in width. The conidia were subglobose (2.5–3.5 μm), and both terminal and intercalary chlamydospores were present (Fig. 4B).

### Molecular confirmation of *T. asperellum* and *T. harzianum*

The ITS region of both isolates was amplified and sequenced. BLASTn analysis confirmed that *Trichoderma* isolates 3 belongs to *T. asperellum* (100% identity and 100% query coverage) and was deposited in GenBank under accession number LC866760.1 and namely SEPA11A. While the *Trichoderma* isolates 35 was molecular confirmed as *T. harzianum* with 98.78–99.82% sequence identity (65–100% query coverage) and deposited in GenBank under accession number LC866759.1 and its namely code is SEPA11A.

**Fig. 5:**
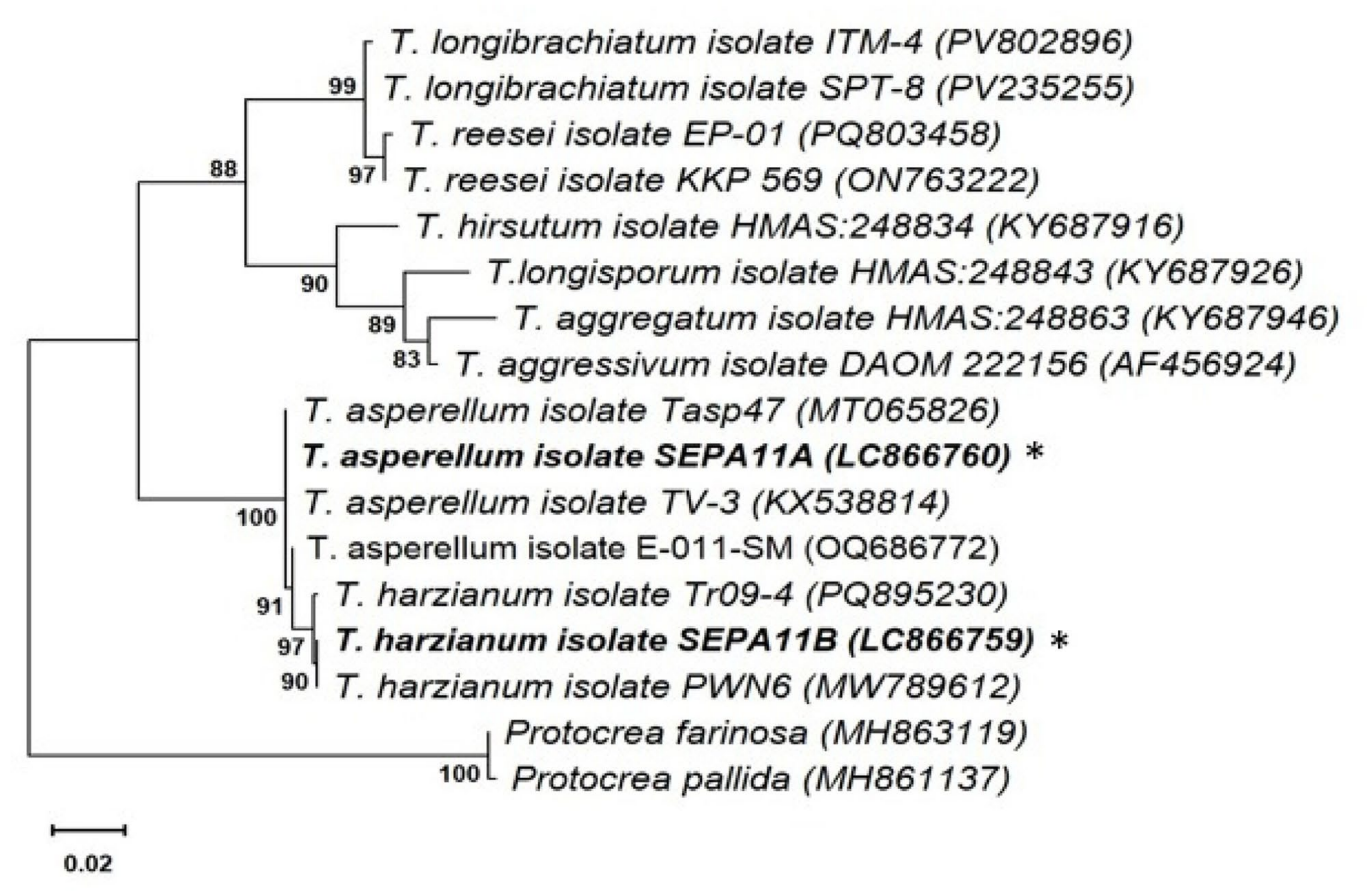
Maximum likelihood phylogenetic tree based on ITS region sequences illustrates the relationship between *T. asperellum* SEPA11A, *T. harzianum* SEPA11B, and other related *Trichoderma* species. The tree was constructed using MEGA12 software with 1,000 bootstrap replicates. *Protocrea* species were included as the outgroup to root the tree. Numbers on the tree branches indicate bootstrap support values, while the scale bar represents a phylogenetic distance of 0.02 nucleotide substitutions per site.

The top ten GenBank matches for each isolate are summarized in Table S1, while a maximum likelihood phylogenetic tree illustrating their relationships with reference sequences is presented in Fig. 5. For further confirmation of species identity, phylogeny analysis was conducted using MEGA12 software employing the maximum likelihood algorithm with 1,000 bootstrap replicates (Fig. 5). Representative *Trichoderma* species were included as reference sequences, and two species of *Protocrea* were used as outgroups. The resulting phylogenetic tree revealed that *T. asperellum* isolate SEPA11A clustered closely with *T. asperellum* isolates Tasp47 (MT065826) and TV-3 (KX538814), supported by a bootstrap value of 100 (Table S1). Similarly, *T. harzianum* isolate SEPA11B clustered with *T. harzianum* isolates Tr09-4 (PQ895230) and PWN6 (MW789612), with bootstrap support values of 97 and 90, respectively (Fig. 5). These results provide strong evidence for the accurate species-level identification of both SEPA11A and SEPA11B.

### Scanning electron microscopy (SEM)

**Fig. 6:**
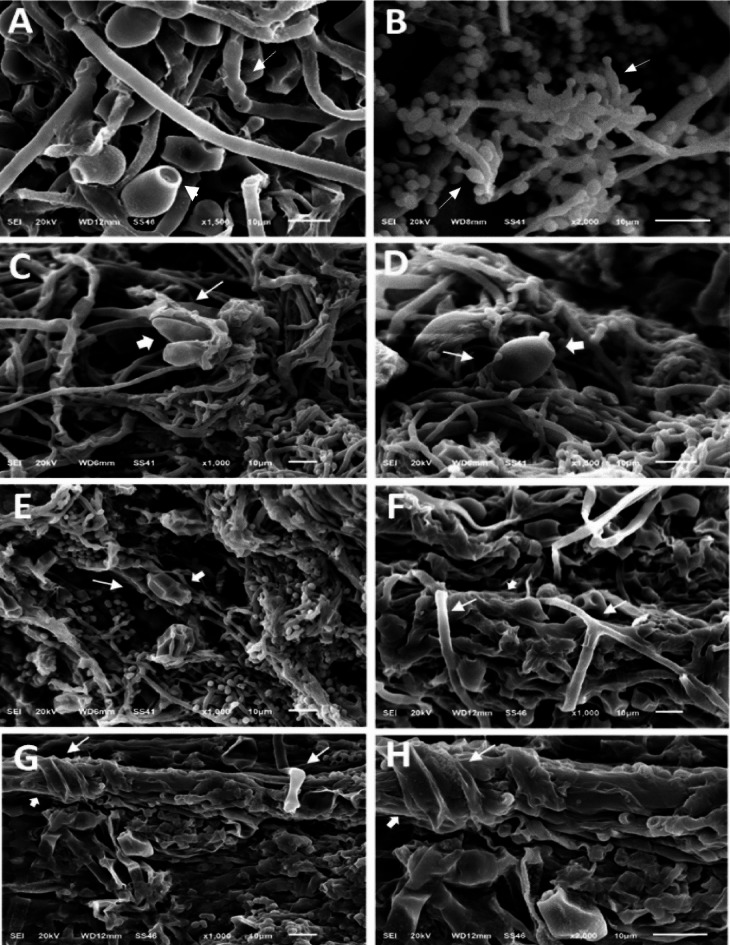
Scanning electron micrographs showing the mycoparasitic interaction of *T. asperellum* SEPA11A and *T. harzianum* SEPA11B with *Curvularia lunata* (SOR7) in dual culture. **A,** Untreated *C. lunata* showing intact hyphae, short conidiophores (arrowheads) and conidia (arrow); **B,** Untreated *Trichoderma* showing characteristic hyphae, branched conidiophores and small conidia (arrows); **C** & **D,** *Trichoderma* conidiophores (arrows) growing over the conidia of *C. lunata* (arrowheads); **E,** *Trichoderma* conidiophores (arrow) in close association with collapsed *C. lunata* conidia (arrowhead); **F**–**H**, Mycoparasitism process, showing *Trichoderma* hyphae (arrows) coiling around and penetrating *C. lunata* hyphae (arrowheads), resulting in hyphal collapse. Scale bar = 10 μm.

**Fig. 7:**
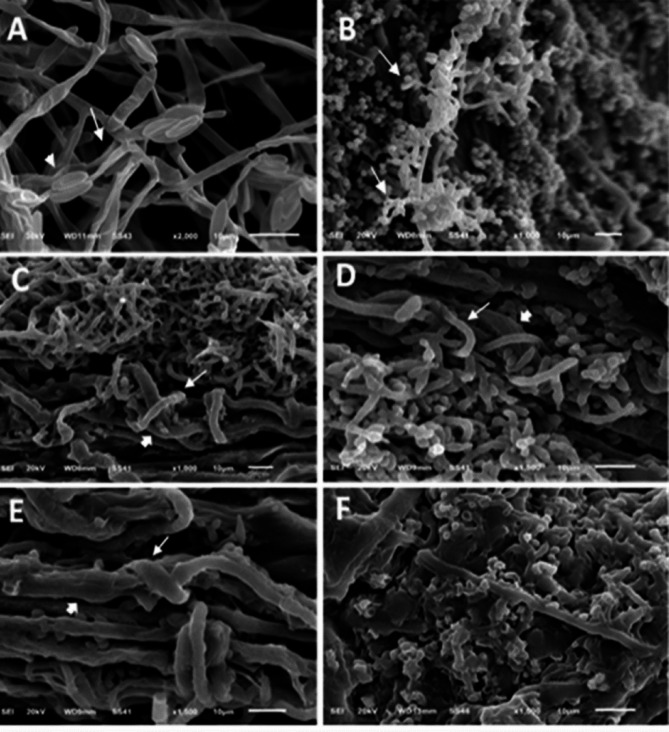
Scanning electron micrographs showing the mycoparasitic activity of *T. asperellum* SEPA11A and *T. harzianum* SEPA11B against *Fusarium verticillioides* (SOR2) in dual culture. **A,** Untreated *F. verticillioides* with normal hyphae (arrows) and conidia (arrowhead); **B**, Untreated *Trichoderma* hyphae and conidia (arrows); **C**–**E**, Mycoparasitism process, where *Trichoderma* hyphae (arrows) coil around and overgrow the hyphae of *F. verticillioides* (arrowheads), causing collapse and deformation of pathogen hyphae. Scale bar = 10 μm.

To gain deeper insights into the antagonistic mechanisms of *T. asperellum* SEPA11A and *T. harzianum* SEPA11B against the target pathogens, scanning electron microscopy (SEM) was employed. SEM micrographs (Figs. 6 and 7) clearly revealed the mycoparasitic interactions between the *Trichoderma* isolates and the pathogens in dual culture assays. The hyphae of *Trichoderma* were observed coiling around and penetrating the hyphae of *C. lunata* (SOR7) and *F. verticillioides* (SOR2), leading to the collapse and deformation of their conidia and mycelia. In contrast, untreated pathogen cultures displayed normal, intact hyphal and conidial structures (Figs. 6 and 7).

### GC-MS analysis of cell-free cultural filtrates of the bioagents

**Fig. 8:**
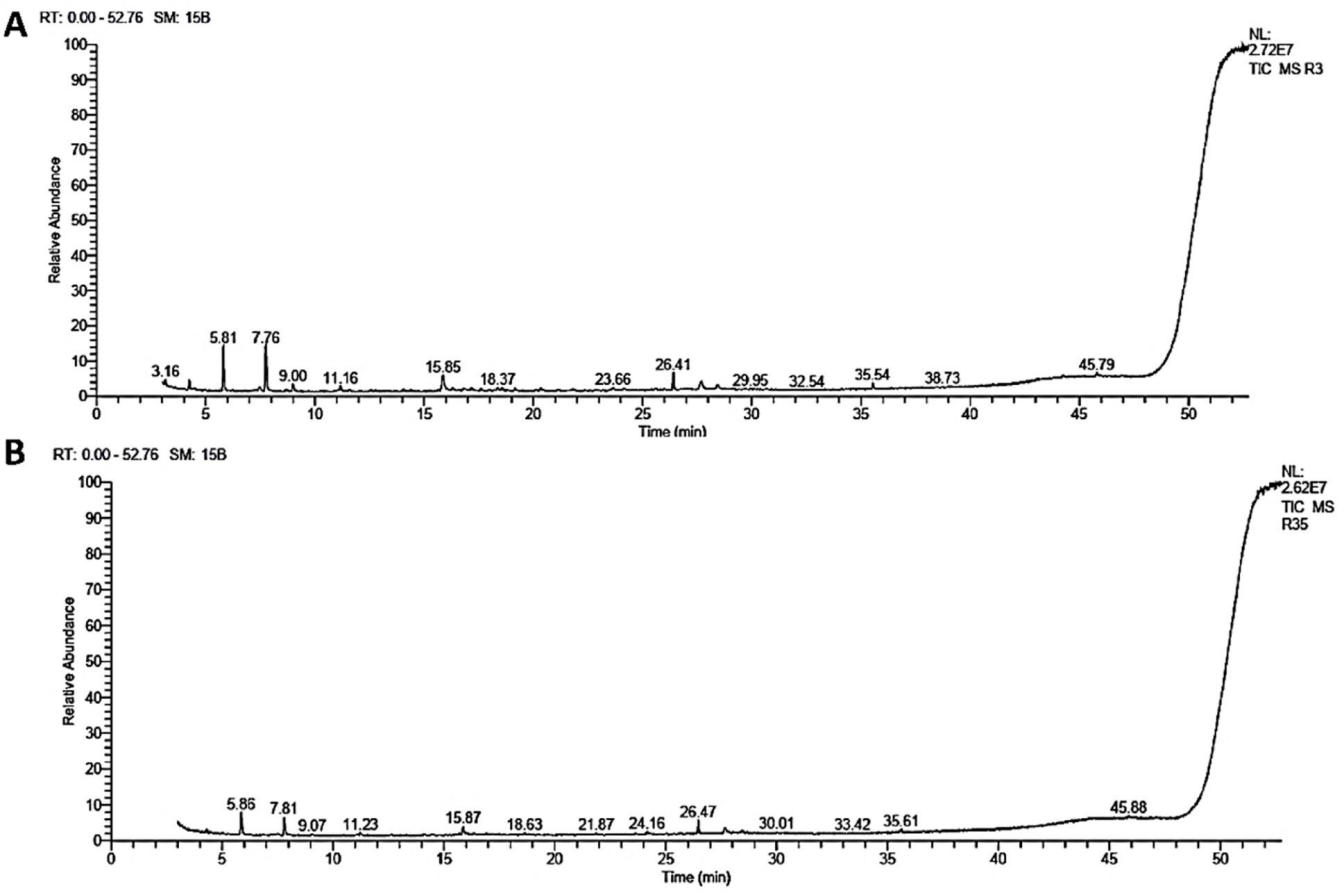
Chromatograms from GC-MS analysis showing the bioactive metabolites present in the culture filtrates of *T. asperellum* SEPA11A (A) and *T. harzianum* SEPA11B (B).

The culture filtrates of *Trichoderma* isolates SEPA11A and SEPA11B were analyzed using GC-MS to determine their metabolite profiles. A total of 55 bioactive compounds were detected, with relative abundances ranging from 1.98 to 23.35% for SEPA11A and from 3.8 to 27.28% for SEPA11B (Table 2; Fig. 8). Of these, 47 compounds were unique to SEPA11A, 22 were unique to SEPA11B, and 14 were shared by both isolates (Table 2).

**Table 2:** Bioactive metabolite compounds identified by GC-MS in the cultural filtrates of *T. asperellum* SEPA11A and *T. harzianum* SEPA11B.

Detected Compounds	Formula	MW	SEPA11A		SEPA11B		Biological activity	Chemical class
			Area %	RT (min)	Area %	RT (min)		
**(S)-(+)−2-Amino-3-methyl-1-butanol**	C_5_H_13_NO	103	23.35	7.76	–	–	Antifungal^44^	Amino alcohols
**[1aR-(1aà**,**2à**,**5á**,**5aá**,**6á**,**8aà**,**9à**,**10aà)]-Hexadecanoic acid**,** 1-(hydroxymethyl)−1**,**2-ethanediyl ester**	C_35_H_68_O_5_	568	2.3	45.8	–	–	–	Fatty acid ester
**14-Octadecenoic acid**,** methyl ester**	C_19_H_36_O_2_	296	2.00	3.16	–	–	Antifungal^45^	Fatty acid
**1-Butene**,** 1-(methylthio)-**,** (E)-**	C_5_H_10_S	102	2.93	7.46	–	–	Antifungal^46^	Thioenol ethers
**1-Monolinoleoylglycerol trimethylsilyl ether**	C_27_H_54_O_4_Si_2_	498	2.3	45.8	–	–	Antifungal, antibacterial and antioxidant^47^	Alkaloid
**2-(Cyclohexylmethylidene) hydrazine-1-carboth ioamide**	C_8_H_15_N_3_S	185	2.93	7.46	–	–	–	Cyclohexyl group
**2-Bromotetradecanoic acid**	C_14_H_27_BrO_2_	306	–	–	9.35	27.68	Antifungal^48^	Fatty acid esters
**2-Myristynoyl pantetheine**	C_25_H_44_N_2_O_5_S	484	4.56	4.52	–	–	–	Organic acids and derivatives
**2-Propanone**,** 1**,**3-dihydroxy-**	C_3_H_6_O_3_	90	23.35	7.76	23.84	7.8	Antifungal^49^	Ketoses
**3-(Prop-2-enoyloxy) pentadecane**	C_18_H_34_O_2_	282	4.56	4.52	–	–	Antifungal and anticonvulsive^50^	Ketone group
**3-(Prop-2-enoyloxy) tridecane**	C_16_H_30_O_2_	254	4.56	4.52	–	–	Anti-bacterial and anti-biofilm^51^	Acrylate group
**3-tert-Butylsulfanyl-3-fluoro-2-trifluoromethyl-a crylic acid methyl ester**	C_9_H_12_F_4_O_2_S	260	2.79	11.17	–	–	–	Acrylic ester
**4-(Prop-2-enoyloxy) tetradecane**	C_17_H_32_O_2_	268	4.56	4.52	–	–	–	Acrylate
**6-Oxa-bicyclo [3.1.0] hexan-3-one**	C_5_H_6_O_2_	98	5.01	8.99	–	–	Antimicrobial and antioxidant^52^	Cyclic ketones
**7-Methyl-Z**,** Z-8**,**10-hexadecadien-1-ol acetate**	C_19_H_34_O_2_	294	–	–	3.8	4.3	–	Fatty acid acetates
**8**,**11-Octadecadiynoic acid**,** methyl ester**	C_19_H_30_O_2_	290	2	3.16	–	–	Antifungal^53^	Fatty acid methyl esters
**9-(3-Fluoro-phenyl)−12-imino-10**,**11-dioxa-tricy clo [6.2.2.0(1**,**6)] dodecane-7**,**7**,**8-tricarbonitrile**	C_19_H_15_FN_4_O_2_	350	3.07	35.55	–	–	–	Polycyclic organic compounds
**9**,**10-Secocholesta-5**,**7**,**10(19)-triene-1**,**3-diol**,** 25-[(trimethylsilyl)oxy]-**,** (3á**,**5Z**,**7E)-**	C_30_H_52_O_3_Si	488	5.16	27.69	3.8	4.3	Anticancer^54^	Steroid family
**9**,**12**,**15-Octadecatrienoic acid**,** 2-(acetyloxy)−1-[(acetyloxy) methyl] ethyl ester**,** (Z**,** Z**,**Z)-**	C_25_H_40_O_6_	436	–	–	3.8	4.3	Antimicrobial and anti-inflammatory^55,56^	Fatty acid derivative
**9-Octadecenoic acid**,** (2-phenyl-1**,**3-dioxolan-4-yl) methyl ester**,** cis**	C_28_H_44_O_4_	444	5.16	27.69	9.35	27.68	Antimicrobial and anti-inflammatory^56,57^	Fatty acid derivative
**à-d-Glucofuranosyl benzenesulfonate**	C_12_H_16_O_8_S	320	3.07	35.55	–	–	–	Organic sulfonates
**Androstane-11**,**17-dione**,** 3-[(trimethylsilyl)oxy]-**,** 17-[O-(phenylmethyl) oxime]**,** (3à**,**5à)-**	C_29_H_43_NO_3_Si	481	2.3	45.8	–	–	Antimicrobial, nti-ainflammatory^57^	Modified steroid molecule
**Benzaldehyde**,** 4-nitro-**,** (6-methyl-1**,**4-diazatricyclo [4.3.1.1(4**,**8)] undec-7 -yliden) hydrazine**	C_17_H_21_N_5_O_2_	327	–	–	9.35	27.68	–	–
**Carbonic acid**,** (ethyl)(1**,**2**,**4-triazol-1-ylmethyl) diester**	C_6_H_9_N_3_O_3_	171	5.01	8.99	–	–	–	Organic carbonates.
**Cyclohexanone**	C_6_H_10_O	98	5.01	8.99	–	–	Antifungal and antibacterial^58,59^	Organic compounds
**Cyclopentanol**,** 1-[2-methyl-3-(methylthio)−2-propenyl]-**,** (Z)-**	C_10_H_18_OS	186	2.93	7.46	–	–	Antimicrobial^60^	Cyclopentanol family
**Cyclopropaneoctanoic acid**,** 2-octyl-**,** methyl ester**	C_20_H_38_O_2_	310	2.00	3.16	–	–	Antifungal against *Sclerotium bataticola*^61^	Fatty acids
**Desulphosinigrin**	C_10_H_17_NO_6_S	4279	3.37	28.45	–	–	Antiparasitic^62^	Glucosinolate
**d-Glucosamine**	C_6_H_13_NO_5_	179	23.35	7.76	23.84	7.8	Antimicrobial^63^	Amino sugars
**d-Glycero-d-ido-heptose**	C_7_H_14_O_7_	210	–	–	10.55	15.86	Antibiotics or immune responses, anti-inflammatory and antiseptic^64^	Carbohydrates (Monosaccharides)
**Dimethylmuconic acid**	CH_10_O_4_	170	3.07	35.55	–	–	Antimicrobial^65^	Dicarboxylic acid class
**Diphenyl sulfone**	C_12_H_10_O_2_S	218	3.07	35.55	–	–	Antibacterial^66^	Organic compounds
**dl-Glyceraldehyde**	C_3_H_6_O_3_	90	21.92	5.81	27.28	5.86	–	Monosaccharide
**dl-Glyceraldehyde dimer**	C_6_H_12_O_6_	180	21.92	5.81	27.28	5.86	–	Organic compound
**D-Mannopyranose**	C_6_H_12_O_6_	180	10.17	15.85	10.55	15.86	Antibacterial^67^	Monosaccharides
**Estra-1**,**3**,**5(10)-trien-17á-ol**	C_18_H_24_O	256	5.16	27.69	–	–	Antifungal^61^	Steroid
**Formic acid**,** 2-propenyl ester**	C_4_H_6_O_2_	86	2.79	11.17	–	–	–	Ester class of organic compounds
**Galacto-heptulose**	C_7_H_14_O_7_	210	–	–	10.55	15.86	Antifungal^68^	C-glycosyl compounds
**Glycine**,** N-[(3à**,**5á**,**7à**,**12à)−24-oxo-3**,**7**,**12-tris [(trimethyl ssilyl) oxy]cholan-24-yl]-**,** methyl ester**	C_36_H_69_NO_6_Si_3_	695	1.98	49.65	5.11	50.24	Antibacterial and antiperspirant^69^	Steroid
**Heptasiloxane**,** 1**,**1**,**3**,**3**,**5**,**5**,**7**,**7**,**9**,**9**,**11**,**11**,**13**,**13-tetradecamethyl**	C_14_H_44_O_6_Si_7_	504	1.98	49.65	5.11	50.24	–	Siloxane compound
**Hexadecanoic acid**,** 1a**,**2**,**5**,**5a**,**6**,**9**,**10**,**10a-octahydro-5**,**5a-dihydroxy-4 -(hydroxymethyl)−1**,**1**,**7**,**9-tetramethyl-11-oxo-1 H −2**,**8a-methanocyclopenta[a] cyclopropa[e] cyclod ecen-6-yl ester**,	C_36_H_58_O_6_	586	2.3	45.8	–	–	–	Fatty acids
**Hexasiloxane**,** 1**,**1**,**3**,**3**,**5**,**5**,**7**,**7**,**9**,**9**,**11**,**11-dodecamethyl**	C_12_H_38_O_5_Si_6_	430	1.98	49.65	5.11	50.24	Antibacterial^70^	Alkaloids
**l-Alanine**,** N-methoxycarbonyl-**,** butyl ester**	C_9_H_17_NO_4_	203	2.93	7.46	–	–	–	Amino acid derivatives
**Methanamine**,** N-hydroxy-N-methyl-**	C_2_H_7_NO	61	21.92	5.81	27.28	5.86	Antifungal against *Candida albicans* and *Aspergillus niger*^71^	Hydroxylamine derivative
**Methyl nitrite**	CH_3_NO_2_	61	–	–	27.28	5.86	Antibacterial and antifungal^72^	Alkyl nitrite
**N**,** N-Dimethyl-O-(1-methyl-butyl)-hydroxylami ne**	C_7_H_17_NO	131	21.92	5.81	–	–	Antifungal^73^	Hydroxylamine derivative
**o-Acetyl-L-serine**	C_5_H_9_NO_4_	147	2.93	7.46	–	–	Anti-inducers^55,56^	L-alpha-amino acids
**Octadecanal**,** 2-bromo-**	C_18_H_35_BrO	346	–	–	3.8	4.3	Antibacterial and antifungal^74^	Bromoalkane
**Octasiloxane**,** 1**,**1**,**3**,**3**,**5**,**5**,**7**,**7**,**9**,**9**,**11**,**11**,**13**,**13**,**15**,**15-hexadeca methyl**	C_16_H_50_O_7_Si_8_	578	1.98	49.65	5.11	50.24	Antimicrobial^75^	Alkaloid
**Oxirane**,** [(2-propenyloxy) methyl]-**	C_6_H_10_O_2_	114	2.79	11.17	–	–	Antifungal^76^	Epoxide and glycidyl ether
**Pentasiloxane**,** 1**,**1**,**3**,**3**,**5**,**5**,**7**,**7**,**9**,**9-decamethyl-**	C_10_H_32_O_4_Si_5_	356	2.87	50.4	–	–	Antibacterial^77^	Organosilicon compounds.
**Phenol**,** 2**,**4-bis(1**,**1-dimethylethyl)-**	C_14_H_22_O	206	8.51	26.41	15.6	26.47	Antibacterial, antifungal, anticancer and antioxidant^48^	Substituted phenols
**Propanal**,** 2**,**3-dihydroxy-**	C_3_H_6_O_3_	90	21.92	5.81	27.28	5.86	Antifungal against *F. oxysporum*^44^	Aldehyde and alcohol functional groups
**Pyracarbolid**	C_13_H_15_NO_2_	217	3.07	35.55	–	–	–	–
**Thioacetic acid**,** S-(3**,**5-dihydroxypentyl) ester**	C_7_H_14_O_3_S	178	5.16	27.69	–	–	–	Thioesters

MW = Molecular weight, RT = Retention time.

In SEPA11A filtrates, the most abundant compounds were (S)-(+)−2-Amino-3-methyl-1-butanol, 2-Propanone, 1,3-dihydroxy-, and d-Glucosamine (23.35%, retention time 7.76 min), followed by dl-Glyceraldehyde, dl-Glyceraldehyde dimer, Methanamine, N-hydroxy-N-methyl-, and N, N-Dimethyl-O-(1-methyl-butyl)-hydroxylamine (21.92%, retention time 5.81 min). In SEPA11B filtrate, the most abundant metabolites (27.28%, retention time 5.86 min) were dl-Glyceraldehyde, dl-Glyceraldehyde dimer, Methanamine, N-hydroxy-N-methyl-, Methyl nitrite, Methyl nitrite, and Propanal, 2,3-dihydroxy-. These were followed by 2-Propanone, 1,3-dihydroxy-, d-Glucosamine, DL-Arabinose, and Mannosamine (23.84%, retention time 7.8 min) (Table 2).

### Interaction between *T. asperellum* SEPA11A and *T. harzianum* SEPA11B

A compatibility assay was performed to assess the potential of *T. asperellum* SEPA11A and *T. harzianum* SEPA11B for co-formulation as bioactive ingredients. Results revealed there was no antagonistic interaction between the two isolates, suggesting they can be combined in a single formulation. This compatibility indicates potential synergistic effects when both isolates are applied together, offering enhanced biocontrol efficacy compared to individual applications.

### Shelf-life assessment of the granular biofungicide formulations (GBF)

The viability of the developed biofungicide formulations was monitored for 14 months under two storage temperatures (5°C and 25 °C). Statistical analysis revealed that the mixed formulation (GPF1 + GPF2) with a water activity (a_w_) of 0.11 showed the highest shelf stability, maintaining viability up to 10 months at 5 °C (3.19 × 10^5^ CFU g^-1^ and 8 months at 25 °C (3.17 × 10^5^ CFU g^-1^, with no significant difference from the initial population (4.71 × 10^5^ CFU g^-1^. All formulas demonstrated improved viability under low water activity of 0.11 and cooler storage conditions, emphasizing the importance of humidity and temperature control for long-term shelf-life (Table 3).

**Table 3:** Effect of storage duration, water activity (a_w_), and temperature on the viability of GBF1, GBF2 and the combined formulation (GPF1 + GPF2).

B		5⁰ C								Mean (A)
	A	0	2	4	6	8	10	12	14	
**GPF1**	**Control**	41 ± 20^c−e^	34.67 ± 11.37^e−i^	26.67 ± 11.55^j−p^	20.87 ± 2.05^p−u^	20.23 ± 0.64^p−w^	14.17 ± 10.21^u−A^	13.87 ± 10.64^v−A^	13.33 ± 11.55^x−A^	**23.10** ^**a**^
	**0.11**	41 ± 20^c−e^	36.67 ± 11.55^d−h^	33.33 ± 11.55^f−j^	26.90 ± 11.09^j−p^	23.57 ± 11.15^k−q^	23.38 ± 1.10^zA^	20.87 ± 13.94^p−u^	16.73 ± 21.26 ^q−z^	**27.81** ^**b**^
	**0.59**	41 ± 20^c−e^	29.17 ± 2.52^i−n^	17.77 ± 12.48^q−y^	13.38 ± 11.51^w−A^	13.36 ± 11.50^w−A^	10.36 ± 1.10^zA^	10.25 ± 0.78^zA^	13.0 ± 1.16^A^	**18.12** ^**b**^
**GPF2**	**Control**	41 ± 20^c−e^	46.67 ± 11.55a-c	40.02 ± 0.06c-f	30.31 ± 1.21 h-k	23.05 ± 10.13 m-s	20.37 ± 2.55^p−v^	13.33 ± 11.55^x−A^	13.0 ± 11.39^y−A^	**28.47** ^**b**^
	**0.11**	41 ± 20^c−e^	45.93 ± 11.28 a-c	36.67 ± 10.85 j-p	26.6 ± 11.43j-p	22.93 ± 10.16 m-s	22.53 ± 8.95^m−t^	16.40 ± 10.28^s−A^	13.9 ± 0.12^zA^	**28.25** ^**b**^
	**0.59**	41 ± 20^c−e^	35.0 ± 2.0e-i	26.87 ± 10.85 j-p	22.33 ± 8.08n-t	20.1 ± 0.35p-x	13.33 ± 11.55 ^x−A^	12.57 ± 8.89^y−A^	10.03 ± 0.12^zA^	**22.65** ^**b**^
**GPF1 + GPF2**	**Control**	41 ± 20^c−e^	45.33 ± 2.31^a−c^	34.33 ± 10.26 ^e−i^	29.33 ± 4.16^i−m^	22.0 ± 6.93^o−t^	15.73 ± 3.23^t−A^	13.80 ± 10.58^v−A^	10.33 ± 1.15^zA^	**26.48** ^**c**^
	**0.11**	41 ± 20^c−e^	49.97 ± 0.12^a^	48.43 ± 3.30^ab^	42.07 ± 7.16^b−d^	36.60 ± 11.43^d−h^	30.14 ± 0.46^h−l^	23.49 ± 11.29^k−r^	18.35 ± 5.80^q−y^	**36.25** ^**c**^
	**0.59**	41 ± 20^c−e^	37.63 ± 8.55^d−g^	32.57 ± 8.89^g−j^	28±0.33 ± 5.77^i−o^	26.67 ± 11.55 ^j−p^	23.30 ± 11.43^l−r^	16.67 ± 11.55^r−z^	10.0 ± 0.00^zA^	**27.02** ^**d**^
**Mean (B)**		41^a^	40.11^a^	32.96^b^	0.003^c^	23.17^d^	19.26^e^	15.69^f^	12.82^g^	
**LSD at 5%**		**A: 2.43, B: 2.29, A×B: 6.88**								
*P* value		< 0.0001								
	**B**	**25⁰ C**								**Mean (A)**
**A**		**0**	**2**	**4**	**6**	**8**	**10**	**12**	**14**	
**GPF1**	**Control**	41 ± 20^a−c^	27.17 ± 11.59^g−i^	23.33 ± 11.55^i−k^	17.68 ± 8.11^l−o^	14.13 ± 11.91^o−r^	6.51 ± 0.86^tu^	0.00^v^	0.00^v^	**16.23** ^**e**^
	**0.11**	41 ± 20^a−c^	27.17 ± 11.59^g−i^	20.00 ± 0.0^k−n^	16.75 ± 11.62^m−o^	16.34 ± 11.02^m−q^	13.68 ± 11.98^o−r^	11.35 ± 3.02^p−t^	60.33 ± 0.31^tu^	**18.98** ^**d**^
	**0.59**	41 ± 20^a−c^	20.50 ± 10.54^k−m^	14.67 ± 9.45^n−r^	10.22 ± 0.67^r−t^	6.26 ± 0.72^tu^	0.00^v^	0.00^v^	0.00^v^	**11.58** ^**f**^
**GPF2**	**Control**	41 ± 20^a−c^	35.93 ± 10.28^c−e^	35.67 ± 9.87^c−e^	26.67 ± 11.55^g−j^	19.97 ± 0.12^k−n^	7.83 ± 2.52^s−u^	0.00^v^	0.00^v^	**20.88** ^**bc**^
	**0.11**	41 ± 20^a−c^	33.39 ± 11.95^d−f^	29.97 ± 0.12^f−h^	22.93 ± 10.16^i−l^	16.67 ± 11.55^m−p^	12.94 ± 10.15^o−s^	12.60 ± 11.11^o−s^	4.44 ± 1.41^u−v^	**21.74** ^**b**^
	**0.59**	41 ± 20^a−c^	26.19 ± 7.96^g−j^	31.00 ± 20^e−g^	19.97 ± 0.12^k−n^	12.93 ± 11.16^o−s^	0.00^v^	0.00^v^	0.00^v^	**15.42** ^**e**^
**GPF1 + GPF2**	**Control**	41 ± 20^a−c^	34.30 ± 11.79^d−f^	31.00 ± 20^e−g^	25.00 ± 8.72^h−k^	16.33 ± 11.02^m−q^	11.00 ± 3.46^q−t^	0.00^v^	0.00^v^	**19.83** ^**cd**^
	**0.11**	41 ± 20^a−c^	45.50 ± 9.59^a^	41.73 ± 6.0^ab^	36.60 ± 11.43^b−d^	29.60 ± 1.39^f−h^	22.57 ± 8.89^i−l^	16.73 ± 11.32^m−o^	9.87 ± 1.55^r−t^	**30.45** ^**a**^
	**0.59**	41 ± 20^a−c^	30.07 ± 0.23^f−h^	22.97 ± 10.28^i−l^	21.53 ± 5.31^j−m^	13.33 ± 11.55^o−r^	0.00^v^	0.00^v^	0.00^v^	**16.11** ^**e**^
**Mean (B)**		41^a^	31.08^b^	26.96^c^	21.93^d^	16.17^e^	8.28^f^	4.52^g^	2.26^h^	
**LSD at 5%**		**A: 1.90, B :1.79, A×B: 5.38**								
**P value**		**< 0.0001**								

*Colony-forming units (CFU) are expressed per gram of formulation. Data are presented as mean ×10^4^ ± SD. Values followed by different letter(s) within the same column indicate significant differences according to Tukey’s test at *P* ≤ 0.05 (*n* = 3).

## Discussion

Ensuring healthy seeds is a prerequisite for optimal crop establishment and yield, that is indicating that getting healthy seed means that presence of good seed with good yield production. The present study revealed a high incidence of seed-borne mycoflora in sorghum, including both saprophytic and pathogenic fungi. A total of 22 fungal species belonging to 16 genera were isolated using both AP and DFB techniques. The AP technique yielded higher fungal diversity, particularly for *A. niger*, *A. flavus*, *A. alternate*, *Fusarium* spp., and *Bipolaris* spp. that have negative effect on seed germination and seed quality due to the mycotoxins/aflatoxins/ochratoxins they produce, which, if consumed by man, cause carcinoma and paralysis^44^. The results are consistent with Mancini et al.^78^ and Parmar et al.^79^, who reported that the AP method generally provides higher sensitivity for detecting a wide range of fungi due to enhanced nutrient availability and clearer colony development. In contrast, the DFB technique is particularly effective for identifying specific, slow-growing pathogens, such as *Fusarium* and *Drechslera*, as it reduces interference from fast-growing saprophytes, although it may increase the risk of seed decay. These findings adequately aligned with the reported results of Khalimi & Anik Leana^44^, who emphasized the critical role of plant quarantine, accurate pathogen detection, and seed health testing to prevent the transmission of seed-borne pathogens. Accordingly, seed health monitoring and fungicidal seed treatments should be integrated into national seed certification programs to mitigate yield losses.

The present study indicated that sorghum seeds are heavily infected with key pathogens, including *Fusarium* sp., *C. lunata*, and *Bipolaris* sp., which may cause seed abortion, seed rot, necrosis, rot, and reduced germination^80^. Raising farmer awareness and implementing field inspections and seed health testing programs are essential for improving seed^81^. These results are consistent with previous reports that pathogenic fungi, including *Fusarium* species, can exist as endophytes within healthy seeds^82,83^. The frequency of endophyte isolation can vary depending on the culture media used^84^ and the type of host tissue^85^. Endophytic fungi often employ specialized colonization strategies to evade host defenses, allowing them to persist asymptomatically within internal tissues^86^. Liao et al.^87^ defined fungal endophytes as asymptomatic microbial partners intimately associated with healthy plant tissues, capable of conferring benefits, co-evolving with the host, and modulating their lifestyle according to plant developmental stage and environmental conditions. In this context, many fungi typically considered pathogens can inhabit seeds and other plant tissues without causing visible disease symptoms, explaining their frequent detection in externally healthy-looking sorghum grains. This dual lifestyle underscores the importance of distinguishing latent endophytic colonization from active pathogenic infection when assessing seed-associated fungi. So, it is the importance of adequate plant quarantine, correct diagnosis of symptoms and/or methods of detection and isolation of such dangerous pathogen which could be transferred through seeds. Therefore, most countries have to examine seed samples carefully and/or have to treat seeds with fungicides.

Endophytic fungi are widely recognized as beneficial microorganisms that colonize internal plant tissues without causing disease symptoms, forming an integral component of the plant microbiome. Among them, *Trichoderma* species are among the most frequently reported endophytic and saprophytic fungi, with strong ecological adaptability, rapid growth, and competitive ability in diverse environmental conditions. In the context of seed transmission ecology, certain endophytes can be vertically transmitted through seeds, enabling early colonization of emerging seedlings and influencing initial plant–microbe interactions^40,88,89^. This seed-borne habit allows *Trichoderma* to establish within host tissues from the earliest stages of development, where it can suppress seed-borne pathogens through mechanisms such as mycoparasitism, antibiosis, and nutrient competition. Collectively, these ecological traits—combining efficient tissue colonization, competitive superiority, and early establishment via seeds—explain why *Trichoderma* remains one of the most reliable and widely studied genera for biological control in sustainable agriculture^90,91^. In this study, *T. asperellum* SEPA11A and *T. harzianum* SEPA11B were formulated into a bioagent and evaluated for antagonistic activity against *C. lunata*, and *F. verticillioides*. Molecular identification based on ITS region sequencing confirmed the identity of both isolates. ITS sequencing remains a cornerstone for fungal taxonomy due to its ability to resolve inter- and intraspecific variation^92,93^. 

*Trichoderma* spp. suppresses pathogens via antibiosis, competition, and mycoparasitism, primarily via secretion of bioactive metabolites such as peptaibols, polyketides, terpenes, gliotoxin, and pyrones^63,94^. GC-MS profiling of cultural filtrates revealed that both isolates produce a diverse array of antimicrobial metabolites. The GC-MS supports the chemical evidence of the bio-agents cell-free culture filtrates by characterization the bioactive compounds produced for the bioagent metabolites activities in their growing in the interaction zone for mycoparasitism process among both *Trichoderma* bioagents and each studied fungal pathogens. The most abundant compound in SEPA11A was (S)-(+)−2-Amino-3-methyl-1-butanol (23.35%), a broad-spectrum antifungal agent reported to disrupt fungal cell membranes^48^. Both isolates also produced 2**-**Propanone, 1,3-dihydroxy- and D-Glucosamine at high levels, which have well-documented antifungal and antibacterial properties^49,63^. SEPA11B uniquely produced 2-Bromotetradecanoic acid (9.35%), an antifungal metabolite effective against soil-borne pathogens^83^. The detection of 2,4-di-tert-butylphenol in both isolates highlights their antioxidant and antimicrobial potential^57^. Collectively, these findings indicate that *Trichoderma* spp. deploy a complex metabolite arsenal that synergistically contributes to pathogen suppression. Further analysis revealed several low-abundance metabolites with specialized biological functions. Cyclohexanone (5.01% in SEPA11A) demonstrated pronounced antibacterial and antifungal activity^95^. In SEPA11B, galacto-heptulose (10.55%) was detected as an antifungal metabolite known to inhibit the microbial shikimate pathway^68^. Additionally, steroidal compounds such as estra-1,3,5(10)-trien-17α-ol (5.16% in SEPA11A) and androstane-11,17-dione (2.3% in SEPA11A) were detected, both have been linked to anti-inflammatory and anticancer activities^96^. Collectively, these findings suggest that *Trichoderma* spp. produce a complex metabolite profile in which major, intermediate, and minor components act synergistically to enhance their biocontrol potential. Comparative profiling of SEPA11A and SEPA11B revealed distinct ecological strategies. SEPA11A synthesized a wider spectrum of antifungal metabolites particularly amino alcohols and fatty acid esters, likely conferring superior activity against soil-borne plant pathogens. In contrast, SEPA11B produced unique brominated fatty acids, indicating a specialized mechanism for niche competition and pathogen suppression^72^. 

The antagonistic potential of these isolates was further confirmed by dual culture assays. SEPA11A and SEPA11B exhibited the highest antagonism index (1.0) against *F. verticillioides* and *C. lunata* SOR7. After eight days of incubation, microscopic observations revealed that both pathogens were completely overgrown by the *Trichoderma* isolates, with a marked reduction in pathogen growth and complete absence of sclerotia formation compared to the control. These findings may be associated with mechanisms such as the secretion of cell wall-degrading enzymes by *Trichoderma* species during mycoparasitic interactions, as reported in previous studies^57^; however, this aspect was not directly investigated in the current work. The observed antagonistic effects of *Trichoderma* isolates may be associated with the secretion of cell-wall-degrading enzymes, such as chitinases and β−1,3-glucanases, during microparasitic interactions with fungal pathogens, as reported in previous studies^97–99^. However, no enzyme activity assays were conducted in the current work, and these mechanisms are suggested based on established literature. Our results showed that SEPA11B actively overgrew both target pathogens, which likely facilitated the formation of boreholes in the host hyphae and subsequent nutrient acquisition. Biocontrol *Trichoderma* isolates are also known to produce diverse toxic secondary metabolites such as pyrones, koninginins, viridin, gliovirin, gliotoxin, and peptaibols, which contribute to pathogen inhibition^91^. The microparasitic mechanism of *T. asperellum* and *T. harzianum* observed here follows a well-documented sequence: Initial pathogen recognition, emergence of conidiophores and compact conidia between the pathogen hyphae, subsequent hyphal coiling around the pathogen, and finally, cell wall lysis through the secretion of hydrolytic enzymes, including β-glucanase, chitinase, and protease^97^. For identifying functionally dominant metabolites and understanding their synergistic modes of action will be a focus of future research, including bioassay-guided fractionation and combinatorial testing *in vitro.* Dual culture assays confirmed the strong antagonistic capacity of SEPA11A and SEPA11B, which completely overgrew *F. verticillioides* and *C. lunata* colonies and inhibited sclerotia formation. The SEM is used to gain deeper insights into the antagonistic mechanisms of *T. asperellum* SEPA11A and *T. harzianum* SEPA11B against the target fungal pathogens. Electron microscopic examination revealed hyphal coiling, lysis, and disintegration of pathogen hyphae, consistent with mycoparasitic behavior. These results are in agreement with previous reports describing *Trichoderma*-mediated hyphal deformation, cytoplasmic leakage, and induction of plant defense responses^100^. 

A successful biocontrol formulation containing fungal propagules should be easy to prepare, maintain its functional properties (viability, germination rate, enzymatic activity) during prolonged storage, and facilitate its application on target organisms^97–99^, this may be related to ISR. So, as a priority for future investigation, since we plan to perform transcriptomic and gene expression profiling to identify the precise ISR signaling cascades and defense-related genes induced following colonization by SEPA11A and SEPA11B. *Trichoderma* species, which are free-living fungi, are known for their ability to promote plant vigor, optimize water use efficiency, and improve the uptake of both macro- and micronutrients. They also enhance photosynthetic performance, increase plant height and stem diameter, improve other agronomic characteristics, and boost overall crop yield^20,98,99,101^. The plant growth-promoting effects of *Trichoderma* are largely attributed to the release of biologically active signaling molecules that diffuse through the soil and act in a manner similar to plant hormones^101,102^. The formulated bioagent exhibited extended shelf life under low-temperature (4–5 °C) and moderate water activity (a_w_ ≈ 0.57) conditions, retaining viability for up to 12 months. These results support the notion that proper formulation and storage conditions are critical for maintaining the efficacy of *Trichoderma*-based bioproducts^96^. Long shelf-life and faster rate of communalization, and success field usage of *Trichoderma* formulation also depending on the characteristics of *Trichoderma* such as, high competency of rhizosphere, highly competition with other soil saprophytes, improving plant growth, easily multiplication of mass, action broad spectrum, excellent control, safety to environment and compatibility between other bioagents^103^. Furthermore, the growing demand for extended shelf-life in biofungicide formulations justifies the selection of semolina as a carrier material in the granular product. Semolina (durum wheat flour) was chosen for two primary functions: First, as a nutrient-rich substrate that supports the survival, viability, and gradual growth of *Trichoderma* during storage and after field application; and second, as a binding agent whose fine particle size and cohesive properties facilitate stable granule formation when combined with inert materials such as kaolin. In addition, cereal-based carriers—including wheat derivatives—are widely recognized in the literature as effective vehicles for fungal biocontrol agents due to their biodegradability, availability, and capacity to preserve microbial viability. Relevant references confirm that this strategy is consistent with established bioformulation approaches^96,97,103,104^. The granular biofungicide is designed for soil delivery, not foliar spray. Granules can be applied directly to soil (e.g., planting rows or root zone) or as a seed coating after light moistening. Spraying is unsuitable, as the dough-based granules are not water-dispersible and would settle. The formulation intentionally maintains a solid granular structure for gradual release and localized *Trichoderma* activity in the rhizosphere^40,91,96,103,104^.

## Conclusion

In this study, two novel endophytic *Trichoderma* strains—*T. asperellum* SEPA11A and *T. harzianum* SEPA11B—were isolated from healthy sorghum seeds and demonstrated strong antagonistic activity against the seed-borne pathogens *F. verticillioides* and *C. lunata*, the causal agents of sorghum stalk rot and leaf spot, respectively. Their biocontrol potential was supported by the identification of 55 bioactive metabolites via GC–MS and confirmed by severe ultrastructural damage to pathogen hyphae observed through scanning electron microscopy, while a granular formulation of both strains maintained high viability for up to 14 months. These findings highlight the dual potential of SEPA11A and SEPA11B as eco-friendly biocontrol agents whose seed-borne endophytic origin confers unique advantages such as systemic protection and vertical transmission, positioning them as promising candidates for sustainable biofungicide development pending future greenhouses and field validation, and large-scale production optimization. including monitoring of colonization dynamics and persistence under different environmental conditions. Also, as a future priority, we plan to use transcriptomic and gene expression profiling to identify the specific ISR signaling pathways and defense genes induced by SEPA11A and SEPA11B colonization, and quantify ISR-related responses and better distinguish between these mechanisms.

## Materials and methods

### Pathogenic fungi

Highly pathogenic isolates of *C. lunata* (SOR7) and *F. verticillioides* (SOR2) were obtained from the Seed Pathology Research Department, Plant Pathology Research Institute, Agricultural Research Center, Giza, Egypt. These isolates were subcultured and maintained on potato carrot agar (PCA) slants (Trafalgar Scientific and Midland Scientific, England), and incubated at 25 ± 2 °C for 5 days to maintain viability and sporulation prior to use in this study.

### Collection of seed samples

Twenty-two healthy-looking sorghum seed samples (500 g each) were collected during September–October 2023 from six Egyptian governorates (Qena, Asyut, Sohag, Luxor, Dakahlia, and Damietta; latitude 26°012′–31°932′ N, longitude 30°098′–33°039′ E. The samples were collected from an area of 50 m × 50 m in a random zig-zag pattern. Samples were labeled, packed in paper bags, transported to the laboratory, and stored at 4 °C until use.

### Isolation of endophytic seed-borne mycobiomes

A total of 22 composite sorghum seed samples were analyzed, which is statistically sufficient. Each sample included 25 seeds per Petri plate with 8 replicates, resulting in 200 seeds examined per sample. Analyses were performed using both agar plate (AP) and deep-freezing blotter (DFB) techniques (ISTA 2007)^105^, ensuring robust detection of endophytic seed-borne fungi across all sampled regions.

Healthy-looking sorghum seeds are characterized by uniform size, weight, and color, typically featuring a smooth, undamaged, and clean surface free from debris, cracks, or discoloration were surface-sterilized by sequential immersion in 70% ethanol (2 min), 2% sodium hypochlorite (1 min), and 75% ethanol (30 s), followed by thorough rinsing with sterile distilled water for 10 min and air-drying under laminar airflow^106^.

For the AP method, 25 seeds were placed on Petri plates containing 20 mL of 2% water agar (8 plates/sample) and incubated for 7 days at 22 ± 2 °C. For the DFB method, 25 seeds were placed on moistened sterile filter papers in sterilized Petri plates (8 plates/sample), incubated at 22 ± 2 °C for 24 h, frozen at −20 °C overnight, and then transferred to a growth chamber under white fluorescent light (12 h light/dark cycles) at 22 ± 2 °C for 5 days. Emerging fungal colonies were transferred to potato dextrose agar (PDA; 200 g potato, 18 g dextrose, 18 g agar, and 1 l distilled water) for purification using single spore and hyphal tip techniques^107^. Pure cultures were examined under a compound microscope (40X magnification) and maintained in a refrigerator (4 °C) for further studies.

### Morphological characterization

Isolated endophytic fungi were characterized based on their macroscopic traits (growth rate, colony color) and microscopic features (conidiophore structure, phialide morphology, conidial size, and presence/absence of chlamydospores) following standard mycological keys^108–113^. Molecular sequencing was not performed due to the large-scale nature and scope of the diversity assessment.

### Dual culture assay for antagonism

Thirty-nine of the endophytic *Trichoderma* spp. from sorghum seeds were selected and screened for their antagonistic activity against both *F. verticillioides* (SOR2) and *C. lunata* (SOR7) using the dual culture technique^114^. A 5 mm agar disc from each *Trichoderma* isolate (5-day-old) was placed 1 cm from the edge of a PDA plate (9 cm diameter), with a 5 mm disc of the pathogen (*F. verticillioides* or *C. lunata*) placed opposite. Controls consisted of each organism grown alone on PDA. All treatments were performed in triplicate, and plates were incubated at 25 ± 2 °C for six days. The percentage inhibition of pathogen growth was calculated relative to the control.

Antagonistic interaction was also rated on a scale of 1 to 5 after 10 days^115^. 1 = *Trichoderma* completely overgrew pathogen; 2 = *Trichoderma* colonized ≥ 2/3 of plate; 3 = both *Trichoderma* and the pathogen colonized half of the PDA plate; 4 = pathogen colonized ≥ 2/3 of plate; and 5 = the pathogen overgrew *Trichoderma*.

### Morphological and molecular identification of the tested *Trichoderma* spp

Among the 39 *Trichoderma* isolates tested, *Trichoderma* isolates 3 and 35 that exhibited the strongest antagonistic antagonistic in vitro potential effects against the target pathogens and were subjected to detailed for cultural, morphological and molecular identification as following:

### Cultural and morphology characterization

For morphological studies of the *Trichoderma* isolates 3 and 35, growth rates were determined on PDA at 25 ± 2 °C under dark conditions. Mycelial discs (5 mm diameter) were placed in 90-mm Petri plates containing PDA, with three replicates per isolate. Colony diameters were measured after 5 days of incubation. required for the mycelium to completely cover the plate surface was recorded, along with colony morphological characteristics, including colony appearance, color, and sporulation. Microscopic observations were performed using a light microscope (Olympus CX41, Olympus Corporation, Japan), and images were captured using AxioVision MTB2004 software (Carl Zeiss AG, Germany). Fungal isolates were morphologically identified based on macroscopic characteristics (growth rate and colony color) and microscopic features, including conidiophore structure, length of phialides, conidial size and the presence or absence of chlamydospores. Identification was carried out according to the taxonomic keys of Domsch et al.^110^, Gams and Bissett^111^, Samuels and Hebber^112^.

### Molecular confirmation

The *Trichoderma* isolates 3 and 35 that cultural and morphology identified as *T. asperellum* and *T. harzianum* were molecularly confirmed, their genomic DNA was extracted using the GeneDirex DNA extraction kit (Cat. No. NA023-0100), following the manufacturer’s protocol. DNA purity and concentration were assessed using a NanoDrop 2000c spectrophotometer (Thermo Scientific, USA). The internal transcribed spacer (ITS) region of each *Trichoderma* isolate was amplified via polymerase chain reaction (PCR) using primers ITS1: (5’-CTTGGTCATTTAGAGGAAGTAA-3’) and ITS4: (5’-TCCTCCGCTTATTGATATGC-3’)^116^. PCR was performed in 50 µL reactions containing 25 µL 2X PCR Master Mix (Thermo Scientific, USA), 1 µL of each primer, 2 µL DNA (50 ng), and 22 µL nuclease-free water. Cycling conditions: 95 °C for 5 min, 35 cycles at 95 °C for 30 s, 50 °C for 30 s, 72 °C for 1 min, followed by 72 °C for 10 min^117^ PCR products were visualized on 1% agarose gel electrophoresis using a 50 bp DNA ladder^118^. Amplicons were purified using the GeneJET Gel Extraction Kit (Thermo Fisher Scientific, USA) and sequenced via Sanger sequencing (Macrogen, Korea)^119^. The resulting sequences were analyzed using the ChromasPro software (Technelysium, Australia) and compared against GenBank entries using BLAST (Basic Local Alignment Search Tool; NCBI) (http://www.ncbi.nlm.nih.gov/BLAST/)^120^. A sequence similarity ≥ 98% was used for species-level identification^121^. Sequences were deposited in GenBank, and a phylogenetic tree was constructed using MEGA12 software with the maximum likelihood method and 1000 bootstrap replications^122,123^.

### Scanning electron microscopy (SEM)

To examine the mycoparasitic interactions, mycelial samples were collected from the interaction zones of dual cultures involving each *T. asperellum* SEPA11A and *T. harzianum* SEPA11B, and the target pathogens, *C. lunata* (SOR7) and *F. verticillioides* (SOR2). These samples represent the interface where *Trichoderma* actively interacts with the pathogen. The specimens were fixed in 2.5% glutaraldehyde and 2% paraformaldehyde in 0.1 M sodium phosphate buffer (pH 7.4) for 12 h at 4 °C, post-fixed in 2% osmium tetroxide for 90 min, and rinsed three times in 0.1 M sodium phosphate buffer containing 0.1 M sucrose. Samples were dehydrated through a graded ethanol series (80, 90, 96, and 100%) and then coated with gold-palladium^40^. Observations were made using a JEOL JSM-6510 LV SEM at 30 kV (Electron Microscopy Unit, Mansoura University, Egypt).

### GC-MS analysis of the bio-agents cell-free cultural filtrates

*Trichoderma* isolates SEPA11A and SEPA11B that have most effective potential antagonism against fungal pathogens were cultured individually in 50 mL potato dextrose broth (PDB) at 25 ± 2 °C for 14 days under static conditions. After incubation, the cultures were homogenized using a blender and filtered through filter paper to remove mycelia. The resulting *Trichoderma* culture filtrates were filtered through filter paper and further clarified by centrifugation at 5000 rpm for 20 min, and then sterilized through 0.45 μm Millipore filters to obtain cell-free culture supernatants^124^. Bioactive compounds were extracted from these cultural filtrates using an organic solvent (Ethyl acetate) at a 1:1, v/v ratio by vigorous shaking for 20 min, and the organic phase was collected and concentrated under reduced pressure using a rotary evaporator at 50 °C. Concentrated extracts were derivatized using standard silylation reagents (BSTFA) to improve volatility and thermal stability for GC–MS analysis (Bruker Scion 536 SQ, Scion, Holland)^125,126^. The derivatized samples were injected in split mode (1 µL) into the Trace GC-TTSQ mass spectrometer (Thermo Scientific 1310 GC, TSQ 9000, Italy) equipped with a TG-5MS capillary column (30 m x 0.25 mm x 0.25 μm). Helium was used as the carrier gas at 1 mL min⁻¹. Compounds were identified by comparing mass spectra with WILEY 09 and NIST 14 libraries^127^.

### Compatibility test between *Trichoderma* isolates SEPA11A and SEPA11B

To assess compatibility, 5 mm discs of SEPA11A and SEPA11B were placed opposite each other (1 cm from plate edges) on PDA. Each isolate grown alone served as a control. Three replicates were used. The cultures were incubated at 25 ± 2 °C for 9 days in the dark. Radial growth and any inhibition zones were recorded^128^.

### Preparation of granular biofungicide (GBF)

*Trichoderma* isolates SEPA11A and SEPA11B were cultured in PDB for 14 days at 25 ± 2 °C, and spore suspensions were adjusted to 3 × 10^6^ spores mL^−^¹ using a hemocytometer under a light microscope. For the preparation of the granular formulations, 1000 mL of each culture filtrate containing fungal conidia was blended and homogenized with semolina (durum wheat flour) and kaolin as carrier materials. Distilled water was incorporated gradually to form a uniform, workable dough. The dough was sheeted using a hand-operated pasta machine, dried at 45 ± 2 °C, ground, and sieved to obtain granules in the size range of 500–2000 μm. Three GBFs were prepared: GBF1 (SEPA11A), GBF2 (SEPA11B), and GBF1 + GBF2 (combined formulation) following the method described by Shabana et al.^104^. The described procedures resulted in a stable granular biofungicide, which is well-suited for subsequent downstream assessment. The intended application method is soil delivery, as opposed to foliar spray. More precisely, the granules can be placed directly into the soil—such as in planting rows or near the root zone. Furthermore, they can also function as a seed coating or dressing: after light moistening to promote attachment to the seed coat, the granules retain their suitability for this use. Their performance will be evaluated in our next phase through downstream greenhouse and open-field studies.

### Shelf-life assessment of GBFs

GBFs were stored under two water activity (a_w_) levels (0.11, and 0.59) and temperatures (4 and 25 °C). Water activity was maintained with saturated salt solutions (LiCl for a_w_ = 0.11, Mg(NO_3_)_2_·6 H₂O for a_w_ = 0.59)^97,129^. Viability was assessed every two months for 14 months by serial dilution plating on PDA and counting colony-forming units (CFU) of fungal propagules. Initial CFU counts were used as reference^130^.

### Statistical analyses

Statistical analyses were performed using CoStat software version 6.4 (CoHort Software, Pacific Grove, CA, USA)^131^. One-way ANOVA was used to evaluate differences among means, and comparisons were made using Tukey’s HSD test. A split-split plot within stripes design was applied to assess the effects of storage duration, water activity (a_w_), and temperature on the viability of GBF1, GBF2, and the combined formulation (GBF1 + GBF2), which their data were expressed as mean ± standard deviation (SD). All statistical tests were conducted at a significance level of *P* ≤ 0.05. All data in the percentage values were arcsine-transformed and Standard Error bars were included only in Figs. 1 and 2.

## Supplementary Information

Below is the link to the electronic supplementary material.

Supplementary Material 1

Supplementary Material 2

## Data Availability

The datasets generated and analysed during the current study are available in the National Center for Biotechnology Information (NCBI) repository, (https://www.ncbi.nlm.nih.gov/nuccore/LC866760.1) and ( https://www.ncbi.nlm.nih.gov/nuccore/LC866759.1).

## Abbreviations

DON: Deoxynivalenol
AP: Agar plate
a_w_: Water activity
BCAs: Biocontrol agents
BLAST: Basic local alignment search tool
CFU: Colony-forming units
DFB: Deep-freezing blotter
GBF: Granular biofungicide
GC–MS: Gas chromatography–mass spectrometry
ISTA: International seed testing association
ITS: Internal transcribed spacer
PCA: Potato carrot agar
PCR: Polymerase chain reaction
PDA: Potato dextrose agar
PDB: Potato dextrose broth
SEM: Scanning electron microscopy
SEPA: Seed pathology and examination analysis
