# Supplementary figures and images for: Laboratory assessment of novel endophytic Trichoderma-based bioformulations for the biological control of sorghum leaf spot and stalk rot diseases
Source: Sci Rep. 2026 Jul 1;16:20156. doi: 10.1038/s41598-026-54927-w (PMC13324313; doi:10.1038/s41598-026-54927-w)

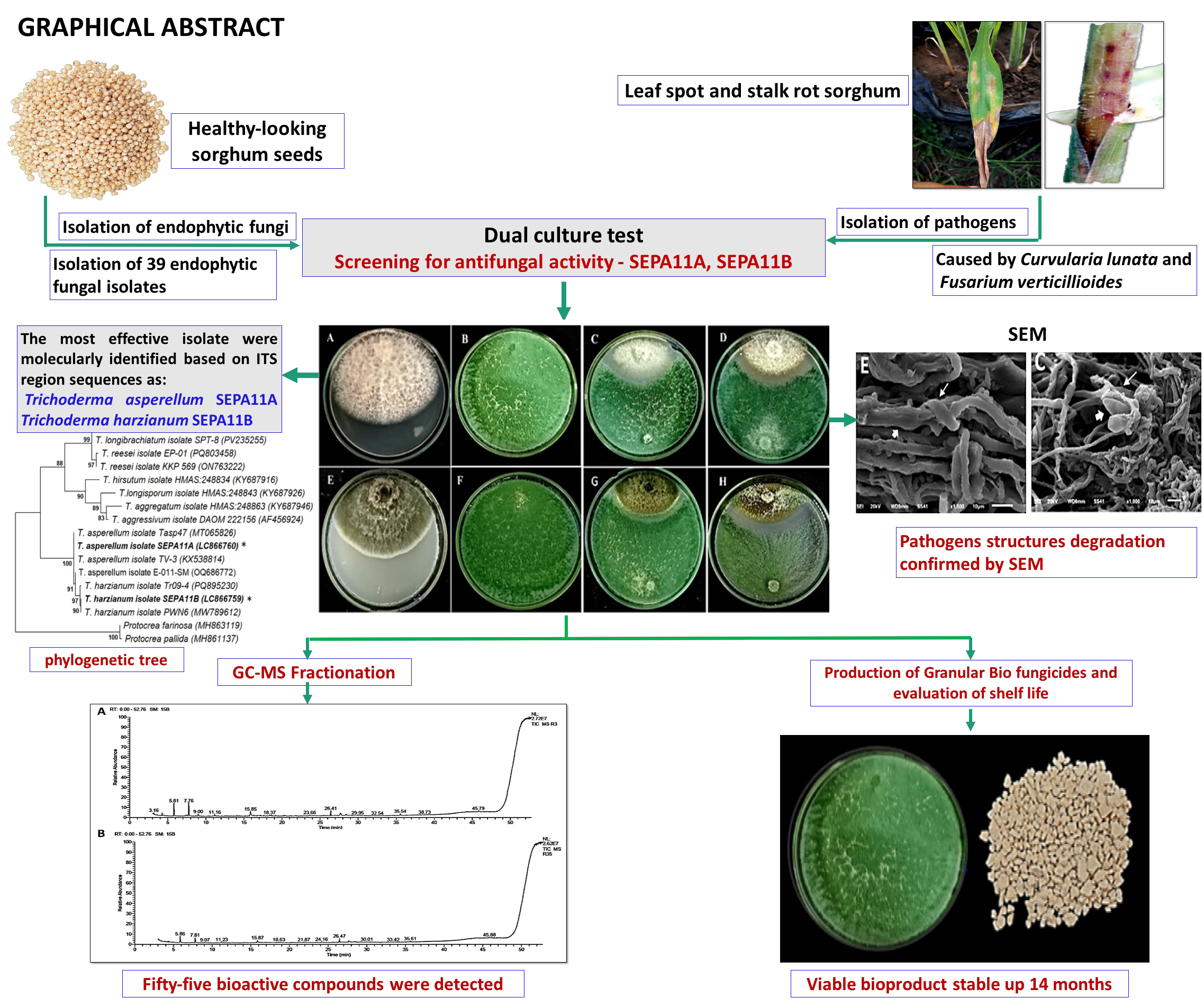

Supplement: Supplementary file 1 — Supplementary Material 1 [file 41598_2026_54927_MOESM1_ESM.docx]
