# Supplementary material for: Laboratory assessment of novel endophytic Trichoderma-based bioformulations for the biological control of sorghum leaf spot and stalk rot diseases
Source: Sci Rep. 2026 Jul 1;16:20156. doi: 10.1038/s41598-026-54927-w (PMC13324313; doi:10.1038/s41598-026-54927-w)
Supplement: Supplementary file 2 — Supplementary Material 2 [file 41598_2026_54927_MOESM2_ESM.docx]

Table S1 Top ten GenBank sequences showing the highest similarity to the internal transcribed spacer (ITS) regions of *Trichoderma* isolates SEPA11A (*T. asperellum*) and SEPA11B (*T. harzianum*), based on BLASTn analysis.

| **Description** | **Query Cover (%)** | **Identities (%)** | **Accession** |
| --- | --- | --- | --- |
| **Isolate (*T. asperellum* isolate SEPA11A)** **LC866760.1** | | | |
| *Trichoderma asperellum* isolate Tasp47 | 100 | 100 | MT065826.1 |
| *Trichoderma asperellum* isolate Tasp46 | 100 | 100 | MT065825.1 |
| *Trichoderma asperellum* strain SD-5 | 100 | 100 | KY807766.1 |
| *Trichoderma asperellum* strain Ct217 | 100 | 100 | PQ787755.1 |
| *Trichoderma asperellum* strain N-8-2 | 100 | 100 | MK720048.1 |
| *Trichoderma asperellum* isolate TV-3 | 100 | 100 | KX538814.1 |
| Trichoderma asperellum isolate Tasp28 | 100 | 100 | KU170995.1 |
| *Trichoderma asperellum* strain Pt9 | 100 | 100 | KU198280.1 |
| *Trichoderma asperellum* isolate TaR5 | 100 | 100 | KT001080.1 |
| *Trichoderma asperellum* strain AF14 | 100 | 100 | JX677934.1 |
| **Isolate (*T. harzianum* isolate SEPA11B)** **LC866759.1** | | | |
| *Trichoderma harzianum* strain PWN6 | 100 | 99.06 | [MW789612.1](https://www.ncbi.nlm.nih.gov/nucleotide/MW789612.1?report=genbank&log$=nucltop&blast_rank=1&RID=WBDA1HCK013) |
| *Trichoderma harzianum* isolate TH3 | 90 | 98.96 | [OP106577.1](https://www.ncbi.nlm.nih.gov/nucleotide/OP106577.1?report=genbank&log$=nucltop&blast_rank=2&RID=WBDA1HCK013) |
| *Trichoderma harzianum* isolate Th-3 | 66 | 99.47 | [MH127468.1](https://www.ncbi.nlm.nih.gov/nucleotide/MH127468.1?report=genbank&log$=nucltop&blast_rank=3&RID=WBDA1HCK013) |
| *Trichoderma harzianum* isolate Th-4 | 65 | 99.64 | [MH127469.1](https://www.ncbi.nlm.nih.gov/nucleotide/MH127469.1?report=genbank&log$=nucltop&blast_rank=4&RID=WBDA1HCK013) |
| *Trichoderma harzianum* isolate Th_8946 | 66 | 99.29 | [OQ318527.1](https://www.ncbi.nlm.nih.gov/nucleotide/OQ318527.1?report=genbank&log$=nucltop&blast_rank=5&RID=WBDA1HCK013) |
| *Trichoderma harzianum* isolate UOM PGPF 03 | 67 | 98.78 | [MN306150.1](https://www.ncbi.nlm.nih.gov/nucleotide/MN306150.1?report=genbank&log$=nucltop&blast_rank=6&RID=WBDA1HCK013) |
| *Trichoderma harzianum* isolate TH01 | 65 | 99.82 | [OP739100.1](https://www.ncbi.nlm.nih.gov/nucleotide/OP739100.1?report=genbank&log$=nucltop&blast_rank=7&RID=WBDA1HCK013) |
| *Trichoderma harzianum* isolate PATB-32 | 65 | 99.64 | [PP830104.1](https://www.ncbi.nlm.nih.gov/nucleotide/PP830104.1?report=genbank&log$=nucltop&blast_rank=8&RID=WBDA1HCK013) |
| *Trichoderma harzianum* isolate KUVKU-TH01 | 65 | 99.82 | [ON319115.1](https://www.ncbi.nlm.nih.gov/nucleotide/ON319115.1?report=genbank&log$=nucltop&blast_rank=9&RID=WBDA1HCK013) |
| *Trichoderma harzianum* isolate Th-2 | 65 | 99.46 | [MH127467.1](https://www.ncbi.nlm.nih.gov/nucleotide/MH127467.1?report=genbank&log$=nucltop&blast_rank=10&RID=WBDA1HCK013) |
